# Selecting Raman spectra filtering based on an exhaustive statistical approach for inline bioprocesses monitoring using *Sf9* insect cells

**DOI:** 10.1007/s00449-026-03301-1

**Published:** 2026-03-26

**Authors:** Milena Miyu Teruya, Júlia Públio Rabello, Luis Giovani Oliveira Guardalini, Fernanda Angela Correia Barrence, Eutimio Gustavo Fernández Núñez

**Affiliations:** 1 Laboratório de Engenharia de Bioprocessos. Escola de Artes, Ciências e Humanidades (EACH), Universidade de São Paulo, Rua Arlindo Béttio, 1000, CEP, São Paulo, SP 03828-000 Brazil; 2 Laboratório de Biotecnologia Viral, Instituto Butantan, Av Vital Brasil, 1500, CEP, São Paulo, SP 05503-900 Brazil

**Keywords:** Artificial neural network, Bioprocess monitoring, Partial least squares, Raman spectroscopy, SARS-CoV-2, Virus-like particles

## Abstract

The spectral preprocessing step of data derived from Raman spectroscopy is important for chemometric models’ calibration, which allows real-time monitoring of pharmaceutical bioprocesses. Thus, the present work aimed to establish, with statistical criteria, the best combination of spectral filters for the biochemical monitoring of the baculovirus/*Sf9* insect cell system. The production of rabies virus-like particles was used as a model. Combining moving window smoothing and offset baseline correction spectral filters demonstrated the highest efficacy in biochemically monitoring the baculovirus/*Sf9* insect cell system using Raman spectroscopic data and Partial Least Squares regression. However, when applying Artificial Neural Network modeling, a different set of filters proved more effective: wavelet denoise spectral smoothing, asymmetric least square baseline correction, standard normal variate normalization, and quadratic first derivative. The simulation results demonstrate that by following these guidelines for spectral preprocessing, cell viability, glucose, lactate, glutamine, glutamate, and ammonium can be satisfactorily monitored in real-time, but not the density of viable cells. The absolute errors for cell viability, glucose, lactate, glutamine, glutamate, and ammonium were lower than 12%, 0.47 g L^− 1^, 8.95 mg L^− 1^, 0.11 g L^− 1^, 0.10 g L^− 1^, 3.21 mg L^− 1^, respectively, which are suitable for bioprocess in-line monitoring and control through a soft sensor.

**Supplementary Information:**

The online version contains supplementary material available at 10.1007/s00449-026-03301-1.

## Introduction

Using spectroscopy tools for pharmaceutical bioprocess monitoring represents a significant advancement for companies in the sector. These techniques encompass various spectroscopies, including UV-Vis, fluorescence, infrared, and Raman. Each of these spectroscopies offers unique advantages for monitoring multiple critical parameters of bioprocesses, such as quantifying biochemicals and cellular concentrations, detecting specific biomolecules, and assessing the structural integrity of biological samples [1].

Among the spectroscopy tools employed in pharmaceutical bioprocess monitoring, Raman spectroscopy stands out for its exceptional capabilities. By leveraging the interaction of laser light with molecular vibrations, Raman spectroscopy provides detailed molecular information in real time. This non-destructive technique allows for analyzing complex biological mixtures without sample preparation, enabling continuous monitoring of bioprocesses. With its high specificity and sensitivity, Raman spectroscopy allows researchers to identify and quantify biomolecules accurately, facilitating process optimization and quality control throughout the bioproduction cycle [2]. By utilizing the scattering of light to probe molecular vibrations, Raman spectroscopy provides valuable insights into the composition and structural changes occurring within pharmaceutical bioprocesses [3].

Specifically, Raman spectroscopy has emerged as a powerful analytical tool for monitoring insect cell bioprocesses. In bioprocesses using this cell host, Raman spectroscopy enabled researchers to monitor key parameters such as cell viability, metabolic activity, and protein expression levels in real time without the sample preparation needed [4]. Researchers can assess insect cells’ metabolic state and viability throughout the process by analyzing specific vibrational modes associated with biomolecules such as proteins, lipids, and nucleic acids [5]. Additionally, by analyzing Raman spectra obtained from bioreactor samples, researchers can quantitatively assess the concentrations of metabolites such as glucose, lactate, and amino acids, providing insights into cellular metabolism and productivity. Moreover, Raman spectroscopy can detect the presence of impurities or by-products in the bioprocess, supporting to ensure the purity and safety of the final product [4, 5].

In conclusion, integrating Raman spectroscopy into pharmaceutical bioprocess monitoring workflows has revolutionized the field by providing valuable insights into complex biochemical processes. These techniques offer unparalleled power for real-time monitoring of key critical parameters, especially in the upstream stage, such as nutrients, metabolites, cell density, and viability, as well as pharmaceutical protein concentrations, enhancing consistency, efficiency, productivity, and economy in biopharma organizations [1]. These outputs are achieved mainly by multiparameter monitoring, which allows for improved control by automated nutrient feeding strategies and process understanding at commercial and lab scales, respectively [2].

The application of Raman spectroscopy within biopharmaceutical processes is fundamentally supported by chemometric models, which are designed to establish precise correlations between spectral data and the biochemical parameters intended for monitoring. Specifically, for animal cell lines, partial least squares (PLS) has been extensively used as a modeling approach. Nevertheless, the application of Raman-based monitoring methods continues to face challenges due to the inherently low signal strength and significant fluorescence interference caused by various biomolecules present in the culture medium. Raman spectroscopy may be integrated with advanced data analysis methodologies and rigorous statistical models, capitalizing on recent advancements in signal processing through machine learning and deep learning algorithms, such as artificial neural networks (ANN) [6].

On the other hand, the importance of spectral preprocessing or filtering in Raman chemometric models can not be overstated, as it directly impacts the accuracy and reliability of the resulting models used in bioprocess monitoring. Spectral preprocessing encompasses a range of techniques that aim to enhance the quality of Raman spectra by removing noise, correcting baseline offsets, and standardizing spectral intensities. Without proper preprocessing, Raman spectra may contain artifacts and variability that can adversely affect the validity of chemometric models, leading to inaccurate predictions and interpretations [7].

One of the key aspects of spectral preprocessing is noise reduction, which involves filtering out unwanted signals while preserving the relevant spectral information. Various noise reduction techniques, such as Savitzky-Golay smoothing, baseline correction, and wavelet denoising, are commonly employed to improve signal-to-noise ratios in Raman spectra [7, 8]. By reducing noise levels, spectral preprocessing enhances signal clarity and facilitates the extraction of meaningful spectral features, thereby improving the robustness and predictive performance of chemometric models [8].

Furthermore, spectral preprocessing plays a key role in addressing spectral variations caused by instrumental factors, sample preparation methods, and environmental conditions. By standardizing spectral intensities and correcting for baseline offsets, preprocessing techniques ensure that Raman spectra are comparable across different samples and experimental conditions. This standardization can help enhance the interpretability and reproducibility of chemometric models. Overall, the careful implementation of spectral preprocessing techniques is essential for optimizing the performance and applicability of Raman chemometric models in various scientific and industrial applications [9].

Moreover, advancements in machine learning and signal processing algorithms have enabled the development of more sophisticated spectral filtering techniques. These approaches leverage statistical models and data-driven algorithms to adaptively filter spectral data based on underlying patterns and structures. By incorporating machine learning techniques such as principal component analysis (PCA), the exactness and trustworthiness of spectral analysis can be improved [5].

In general, the evolution of spectral filtering approaches reflects a dynamic interplay between experimental insights and computational innovations. By integrating experimental validation with computational optimization, researchers continue to refine and expand the repertoire of spectral filtering techniques, ultimately enhancing the quality and interpretability of spectroscopic data across various scientific disciplines and applications. However, to the best of our knowledge, there is no standardized strategy for performing spectral processing in systems that use insect cells, which utilize specific culture media. In Bioprocess in general, the predominant approach is trial and error, using a process manually intensive [2]. Accordingly, this study was undertaken to rigorously optimize the Raman spectra filtering protocol through an exhaustive statistical methodology, to advance inline bioprocess monitoring within the baculovirus/insect cell system.

##  Materials and methods

### Cell lines and culture media

*Sf9* cells, maintained in suspension (ATCC 1711), were cultured in Sf-900™ III serum-free medium (Thermo Fisher Scientific, USA) for the propagation of baculovirus and production of rabies virus-like particles (rabies VLP). For viral titration assays, *Sf9* ET cells (Easy Titer), kindly provided by Professors Ralph Hopkins and Dominic Esposito from the National Cancer Institute (Frederick, MD, USA), were cultured in Sf-900™ III medium supplemented with 2.5% (v/v) fetal bovine serum (FBS, HyClone^®^, Cytiva, USA). Additionally, *Sf9* cells were grown as monolayers for cell transfection experiments in 25 cm² culture flasks.

### Creation and preservation of the recombinant baculoviruses

Initial viral stocks were produced by transfecting 10 µg of recombinant bacmids (Bac-to-Bac™ Baculovirus Expression System, Gibco/ThermoFisher Scientific, catalog number: 10359016, CA, USA), each containing either the gene for the rabies virus matrix protein (M) or glycoprotein (G), complexed with cationic liposome (Cellfectin II^®^, Invitrogen, Massachusetts, USA), into 5 × 10⁶ *Sf9* cells using a 25 cm² culture flask in Sf-900™ III medium. The cells were cultured for 96 h. The supernatant was clarified and stored at 4 °C (avoiding contact with light), establishing passage batch 1. To enhance the yield of infectious baculoviruses, two consecutive infections were conducted in Schott flasks using a 0.1 pfu/cell multiplicity of infection at 1 × 10⁶ cells/mL cell density, with a rotational speed of 100 rpm for 96 h. Both culture supernatants were handled and conserved under the same conditions as batch 1, resulting in batches 2 and 3.

### Cell inoculum for bioreactor assays

To prepare the inoculum for bioreactor runs, cells were initially thawed from a working cell bank (with fewer than 20 passages since the initial thawing of cells provided by ATCC). A series of three passages was conducted, starting with an initial cell density of 0.5 to 1 × 10⁶ cells/mL. Cultures were maintained in 100 mL shake flasks (Schott AG™, Mainz, Germany) with a working volume of 20 mL, using a rotating shaking incubator (Innova 4000, New Brunswick Scientific, Edson, NJ, USA) set at 28 °C with 100 rpm orbital stirring. Cells were collected in the middle of the exponential phase (4–5 × 10⁶ cells/mL and > 90% viability) for each passage.

###  Bioreactor runs

Five batch operation experiments were conducted in a 2 L Bioflo 110 bioreactor (New Brunswick Scientific, Edison, NJ) at 28 °C, using a marine propeller impeller at 80 rpm and a working volume of 1 L. The dissolved oxygen tension (DOT) was regulated and maintained at 30% air saturation by adjusting the inlet gas mixture composition. The specific gas flow rate was set at 0.2 vvm (volume of gas per volume of liquid per minute). The Bioflo 110 was connected to a computer with custom software developed in LabVIEW (National Instruments, Texas, USA), which continuously recorded variables such as pH, temperature, stirring speed, and DOT in real time. Each experiment lasted approximately 120 h.

Samples were collected up to three times a day to assess cell density, viability, and metabolic activity. Additionally, recombinant baculovirus titration and immunochemical and electron microscopy characterization of virus-like particles (not included in this work) were performed less frequently to monitor and confirm virus infection, protein expression, and VLP production [10].

In the experiment with uninfected insect cells, *Sf9* cells were inoculated in pre-warmed serum-free Sf-900^TM^ III medium to an initial density of 5 × 10⁵ cells/mL (Run 1, 12 samples). For the propagation of recombinant baculovirus carrying the rabies glycoprotein gene (rBV-G), *Sf9 *cells at an initial density of 7.4 × 10⁵ cells/mL were infected with rBV-G at a multiplicity of infection (MOI) of 0.1 pfu/cell, with the time of infection (TOI) around 24 h when the viable cell density was close to 1.0 × 10⁶ cells/mL (Run 2, 12 samples).

Experiments for propagating recombinant baculovirus with the rabies matrix protein gene (rBV-M) involved infecting *Sf9 *cells at an initial density of 7.2 × 10⁵ cells/mL with rBV-M at an MOI of 0.1 pfu/cell, with a TOI around 36 h (Run 3, 13 samples). Additionally, an assay was conducted to propagate rBV-M by deliberately infecting the cell inoculum with the recombinant baculovirus to increase sample size and enhance the model’s robustness, allowing the calibrated models to manage common disturbances in this biopharmaceutical process (Run 4, 13 samples).

For the experiment aimed at producing rabies VLP, *Sf9* cells at an initial density of 5 × 10⁵ cells/mL were co-infected at a TOI of approximately 24 h with rBV-G and rBV-M at MOIs of 3 pfu/cell and 2 pfu/cell, respectively (Run 5, 15 samples). The MOIs’ selection was based on prior research on generating rabies VLP containing proteins G and M [11].

A total of 65 samples were collected to form the database for calibrating the chemometric models.

### Cell density and viability

Total cell counts were determined using a light microscope and an improved Neubauer counting chamber (Precicolor, HBG, Giessen-Lützellinden, Germany). Samples were suitably diluted with phosphate-buffered saline (PBS, pH 7.0). Concurrently, cell viability and viable cell counts were assessed using the trypan blue exclusion method.

### Analysis of nutrients and metabolites

Cell metabolism was monitored offline during the five bioreactor experiments, following established protocols. Five-milliliter samples were collected at various intervals throughout the batches. These samples were clarified by centrifugation at 750 × *g* for 4 min, then the supernatants were filtered through a 0.22 μm filter and frozen at -20 °C until nutrient and metabolite analysis. The concentrations of glucose, lactate, glutamine, and glutamate in the supernatant samples were measured using a YSI 2950D-3 Biochemistry Analyzer (YSI Life Sciences, Yellow Springs, OH, USA). Additionally, ammonium levels were quantified using ion-selective electrode on the same bioanalyzer.

###  Real-time monitoring using Raman spectroscopy and the collection of spectral data

Inline Raman spectra were obtained using a stainless-steel immersion probe (12.7 mm in diameter) with a sapphire window, connected to one of the four channels of the RXN2 multichannel Raman spectrometer (Kaiser Optical Systems Inc., KOSI, Ann Arbor, MI, USA). The spectrometer was equipped with a 785 nm laser source, delivering approximately 200 mW of power at the sample. Raman spectra were collected and recorded using IC Raman 4.1 software (Mettler Toledo Autochem, Columbia, MD), which also handled cosmic ray elimination and dark signal subtraction. Spectra were acquired continuously every 30 s within the spectral range of 100–3425 cm⁻¹ at a resolution of 1 cm⁻¹. For model calibration, the average spectrum was calculated using six spectra collected immediately before the sampling moment [12].

### Spectral data filtering assessment

Predictive models were developed using Partial Least Squares (PLS) and Artificial Neural Networks (ANN). These regression models correlated inline Raman spectra (X) with offline reference measurements of growth (cell density and viability) and biochemical variables (nutrients and metabolites) (Y). The whole Raman shift range (100–3425 cm⁻¹) was analyzed. The spectra underwent pre-processing (filtering) before modeling with the two regression techniques, utilizing all 480 possible combinations of smoothing (6), baseline correction (5), normalization (4), and other (4) spectral filtering approaches, along with 10 repetitions of the raw spectral data shown in Table 1. SIMCA^®^ version 17 (Sartorius Stedim Biotech, Göttingen, Germany) was employed for both the pre-processing of spectra and Principal Component Analysis (PCA), which reduced the dimensions of the original and pre-processed spectral data into principal components (PC) used in ANN regression. Moreover, the cumulative fraction of Y variation explained up to a specific component (R²cum) and the cumulative fraction of Y variation predicted by the X model up to the same component according to cross-validation (Q²cum) were obtained using the same software.

**Table 1:** Assessed general and specific spectral filtering approaches in this work before applying PLS and ANN regression techniques.

General spectral filtering	Specific spectral filtering
Smoothing	1. Savitzky-Golay (second-order polynomial, 15 points in each sub-model)
	2. Exponentially Weighted Moving Average (EWMA, symmetric)
	3. Wavelet Denoise Spectral (WDS, mean detrend mode Daubechies wavelet function, 4th wavelet order)
	4. Moving window (MW, median moving window type, window size = 15)
	5. Asymmetric least square (AsLS, smoothing factor = 10000, asymmetry factor = 0.001)
	6. None
Baseline correction	7. Row-center
	8. Offset (Lowest type)
	9. Linear
	10. AsLS (AsLS, smoothing factor = 10000, asymmetry factor = 0.001)
	11. None
Normalization	12. Standard normal variate (SNV)
	13. Peak height
	14. Peak area
	15. None
Other	16. Multivariant scattering correction (MSC)
	17. First quadratic derivate
	18. Second cubic derivate
	19. None

### Modeling techniques

**Fig. 1:**
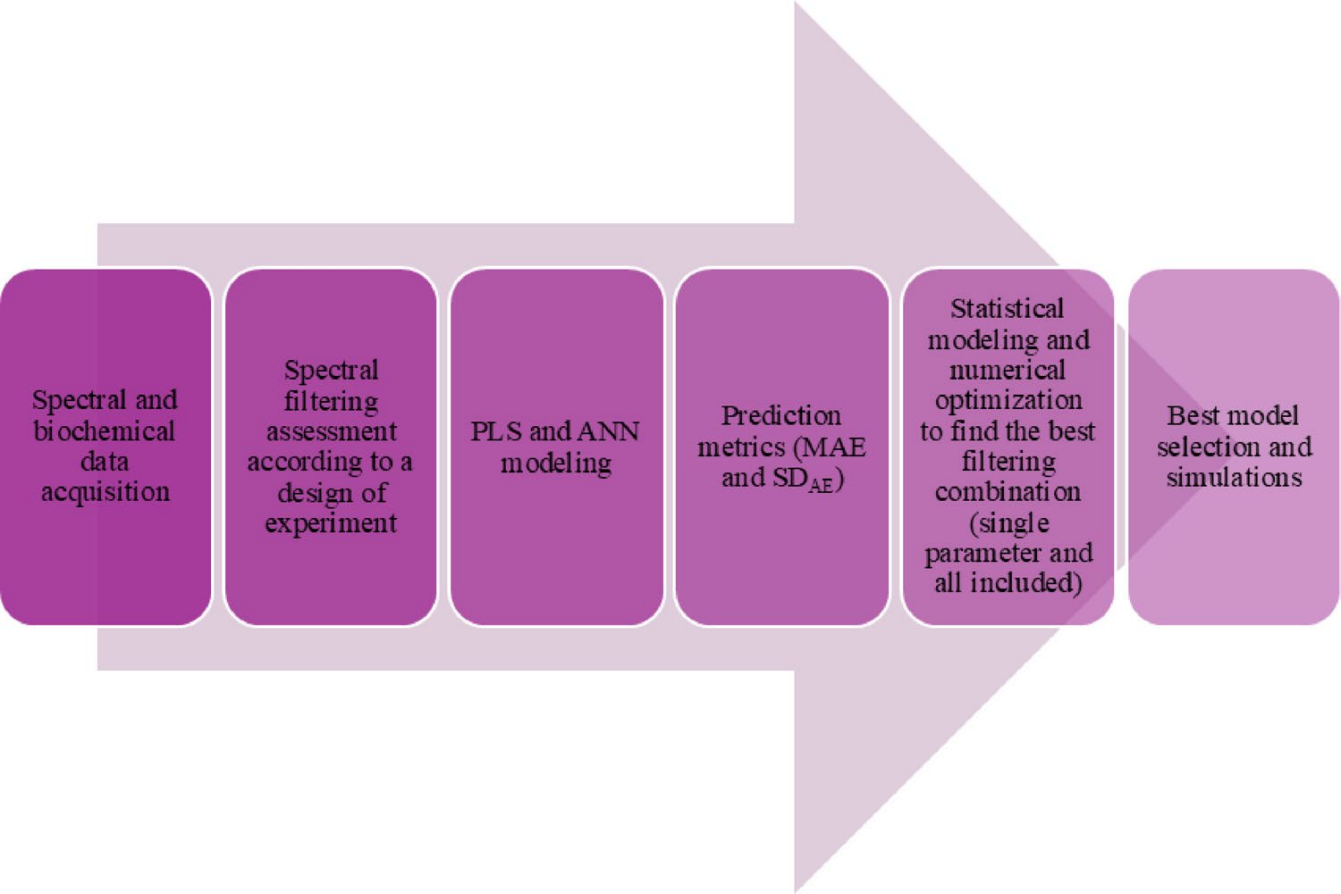
Workflow for selecting Raman spectra filtering based on an exhaustive statistical approach.

Spectral data after filtering assessment were used to calibrate prediction models (PLS and ANN) for biochemical parameters, and then prediction metrics were calculated. Subsequently, statistical models were fitted to connect prediction metrics (dependent variables) with general spectral filtering approaches (independent variables). Finally, the best combination of general spectral filtering approaches was determined by statistical optimization for each biochemical parameter individually and all combined, in the last case using a desirability function. The overall workflow is represented in Fig. 1, and more details can be found in the following two sections.

#### Partial Least Squares (PLS)

Modeling using PLS was performed with SIMCA^®^ version 17, which also facilitated the comparison of the predictive capacity of each generated model according to 480-spectrum filtering for every biochemical parameter. The spectral data were randomly divided into two sets, with 80% allocated for calibration and 20% for validation using the Calibration Wizard tool. A cross-validation procedure was implemented using seven groups to improve model fit. The selected metrics to assess PLS correlation were the mean of absolute error (MAE) (Eqs. 1 and 2), and the Standard Deviation of the Absolute Errors (SD_AE_) (Eq. 3).

1 $$\:\mathrm{M}\mathrm{A}\mathrm{E}=\frac{\sum\:_{\mathrm{i}=1}^{\mathrm{n}}{\mathrm{A}\mathrm{E}}_{\mathrm{i}}}{\mathrm{n}}$$

2 $$\:\mathrm{A}\mathrm{E}=\left|{\mathrm{y}}_{\mathrm{o}\mathrm{b}\mathrm{s}}-{\mathrm{y}}_{\mathrm{p}\mathrm{r}\mathrm{e}\mathrm{d}}\right|$$

3 $$\:{\mathrm{S}\mathrm{D}}_{\mathrm{A}\mathrm{E}}=\sqrt{\frac{\sum\:_{\mathrm{i}=1}^{\mathrm{n}}\left({\mathrm{A}\mathrm{E}}_{\mathrm{i}}-\mathrm{M}\mathrm{A}\mathrm{E}\right)}{\mathrm{n}}}$$

Where:

$\:\mathrm{A}\mathrm{E}$: is the absolute error.

$\:\mathrm{n}$: is the number of samples.

$\:\mathrm{i}$: is the number of the sample.

$\:{\mathrm{y}}_{\mathrm{o}\mathrm{b}\mathrm{s}}$: is the observed value of the biochemical parameter.

$\:{\mathrm{y}}_{\mathrm{p}\mathrm{r}\mathrm{e}\mathrm{d}}$: is the predicted value by the model of the biochemical parameter.

The best one among the 480 spectrum filtering alternatives using the PLS regression technique for each biochemical parameter, and all these combined (overall), was performed in Stat-Ease 360 Software (Stat-Ease Inc, Minneapolis, USA). The response variables were $\:\mathrm{M}\mathrm{A}\mathrm{E}\:$ (Level 5 of importance) and $\:{\mathrm{S}\mathrm{D}}_{\mathrm{A}\mathrm{E}}$ (Level 4 of importance) and their minimum were sought using the optimization algorithm based on the desirability function, with 95% statistical significance (α = 0.05).

#### Artificial neural network (ANN)

The selected architecture for developing models through nonlinear regression by ANN was the multilayer perceptron (MLP). The input data consisted of the principal components (PC) scores of the spectral data, which were divided into three subsets: 70% for training, 15% for testing, and 15% for validation. The modeling process utilized a single hidden layer, with the number of neurons in this layer (N_HN_) explored from 1 to *N* + 1, where N represents the number of input neurons. This approach follows the criterion that N_HN_ ≤ *N* + 1 is suitable for the hidden layer topology. The output layer comprised a single neuron corresponding to each dependent variable in the regression processes.

Five activation functions were employed for both the hidden layer and output layer neurons: identity, logistic sigmoid, hyperbolic tangent, exponential, and sine. Consequently, 25×(*N* + 1) networks were trained for each model. The Automated Network Search (ANS) algorithm in Statistica™ 14.0.1 (TIBCO Software Inc., Palo Alto, CA, USA) was used to adjust the models with these parameters. ANS is a search algorithm that designs and tests multiple neural networks for prediction problems, selecting the networks that show the highest correlation between target and output variables.

To identify models with the best predictive capabilities for dependent variables among 480 spectral filtering approaches, the mean absolute error (MAE) was used as a metric alongside the standard deviation of absolute errors ($\:{\mathrm{S}\mathrm{D}}_{\mathrm{A}\mathrm{E}}$), similar to the PLS regression technique. The method for determining the optimal spectral filtering combination for each biochemical parameter and overall in ANN modeling was the same as that used for the PLS regression technique.

### Statistical comparison between the best spectrum filtering approach in each assessed modeling technique

To determine if there was a statistically significant difference between the best PLS and ANN models, a one-tailed paired Student’s t-test was performed. This test compared the means of two paired groups with α = 0.05, using the AE as the comparison parameter.

The relative error (RE, Eq. 4) was also considered to compare the best spectral filter combination in both modeling techniques using either individual biochemical parameters or overall optimization approaches. The average and its 95% confidence interval were also calculated from the sample set in a defined filter combination.

4 $$\:\mathrm{R}\mathrm{E}\:\left(\%\right)=\frac{\left|{\mathrm{y}}_{\mathrm{o}\mathrm{b}\mathrm{s}}-{\mathrm{y}}_{\mathrm{p}\mathrm{r}\mathrm{e}\mathrm{d}}\right|}{{\mathrm{y}}_{\mathrm{o}\mathrm{b}\mathrm{s}}}\bullet\:100$$

### Simulation of biochemical parameters’ profiles

The most effective chemometric models were validated using hourly Raman spectral data that had not been previously included in the model definitions. The detailed procedure to perform this task was recently published [12].

## Results & discussion

### Models’ fitting for MAE and SD_AE_ using PLS for each biochemical parameter

To optimize the choice of the combination of spectral filters that minimizes the $\:\mathrm{M}\mathrm{A}\mathrm{E}\:$and $\:{\mathrm{S}\mathrm{D}}_{\mathrm{A}\mathrm{E}}$ for each biochemical parameter individually or considering all simultaneously, statistical modeling using PLS was initially performed for these two predictive quality indicators of regression models (Supplementary material: Tables 1 and 2). The models presented were related to coded independent variables and derived from a complete factorial design. The negative sign of the filters or the combination of them indicates that they reduce $\:\mathrm{M}\mathrm{A}\mathrm{E}\:$and their dispersion $\:{\mathrm{S}\mathrm{D}}_{\mathrm{A}\mathrm{E}}\:$depending on the variable under analysis, whether it has a direct relation with the factors. In some cases, the relation was inverse because the response variable transformation was performed to improve the fitting quality of the model.

**Table 2:** Best PLS-generated models for overall and individual optimization.**

Parameter	Optimization analysis	Spectra preprocessing approach				Number of LV***	MAE ± SD_AE_
		Smoothing	Baseline correction	Normalization	Other		
Xv (10^5^ cells/mL)	Overall	MW	Offset	-	-	8	5.75 ± 5.37
	Individual	EWMA	Linear	-	MSC	9	5.43 ± 5.08
CV (%)	Overall	MW	Offset	-	-	7	11.94 ± 9.22
	Individual	MW	-	-	-	7	^*^10.08 ± 8.54
Gluc (g/L)	Overall	MW	Offset	-	-	4	0.46 ± 0.42
	Individual	SG	Offset	-	1^st^ Der. Quad.	1	0.47 ± 0.43
Lac (10^− 3^ g/L)	Overall	MW	Offset	-	-	8	8.95 ± 9.88
	Individual	WDS	AsLS_C	SNV	1^st ^Der. Quad.	11	8.26 ± 8.98
Gln (g/L)	Overall	MW	Offset	-	-	4	0.11 ± 0.09
	Individual	WDS	AsLS_C	SNV	2^nd^ Der. Cub.	9	0.11 ± 0.09
Glu (g/L)	Overall	MW	Offset	-	-	8	0.10 ± 0.09
	Individual	WDS	AsLS_C	-	-	12	^*^0.09 ± 0.10
NH_4_^+^ (10^− 3^ g/L)	Overall	MW	Offset	-	-	9	3.30 ± 2.89
	Individual	MW	Offset	SNV	-	9	3.21 ± 2.68

^*^MAE from individual optimization analysis is lower than that from overall optimization. Finding defined by t-test

^**^A total of 480 combinations of spectral filters, derived from a database of 65 samples collected across five batch experiments, were used to calibrate the MAE and SD_AE_ models

^***^The latent variables (LVs) were determined using the Calibration Wizard tool of SIMCA 18 (trial version), which performs data preprocessing and k-fold cross-validation

The spectral filters for normalization showed the lowest relevance (non-statistical significance, *p* > 0.05) among the considered filter categories for $\:\mathrm{M}\mathrm{A}\mathrm{E}$ and $\:{\mathrm{S}\mathrm{D}}_{\mathrm{A}\mathrm{E}}$ PLS models in the great majority of the biochemical parameters (Table 1 and Supplementary material: Tables 1 and 2). Besides, the combination of smoothing and baseline correction, as well as smoothing and other spectral filters, was the most significant for improving both prediction metrics in PLS (Table 1 and Supplementary material: Tables 1 and 2).

### Choosing the best combination of spectral filters for each biochemical parameter independently using PLS

When the filter combination optimization was performed for each parameter individually, it was not possible to define a recurrent spectral filters’ combination for smoothing, baseline correction, normalization, and others (Table 2). The predictive capacity of all models in this work was equal to or superior (lower magnitude errors) to that reported for inline monitoring of the same biopharmaceutical process using a similar spectral range (Absolute errors for Xv, CV, Gluc, Lac, Gln, Glu, NH_4_^+^ were 5.16 $\:\times\:\:$10^5^ cells/mL, 13.89%; 0.71 g/L; 10.0 $\:\times\:$10^− 3^ g/L; 0.12 g/L; 0.15 g/L; 5.0 $\:\times\:$10^− 3^ g/L, respectively) [13]. When compared to the values reported for the spectral range 400–1850 cm^− 1^, which includes molecular fingerprints for glucose, lactate, glutamine, and glutamate, the results were similar [12]. Thus, the intact Raman spectra could be used for a quantitative analysis equivalent to spectral ranges more favored in terms of chemical information.

The errors obtained in the present work for viable cell density, glucose, and lactate were lower or similar to those reported for systems using mammalian cells where an experimental design approach was also applied [4] However, the optimal spectral filtering combinations were different. This could be explained mainly by the discrepancies between the culture medium and the cell host, which can interfere with the fluorescence background, one of the weaknesses of Raman spectroscopy [2]. Dong and collaborators used the Savitzky–Golay first-order derivative to reduce baseline shift and enhance resolution. Then, SNV was employed for scattering correction [4].

### Selection of the best combination of spectral filters for all biochemical parameters analyzed simultaneously using PLS

**Fig. 2:**
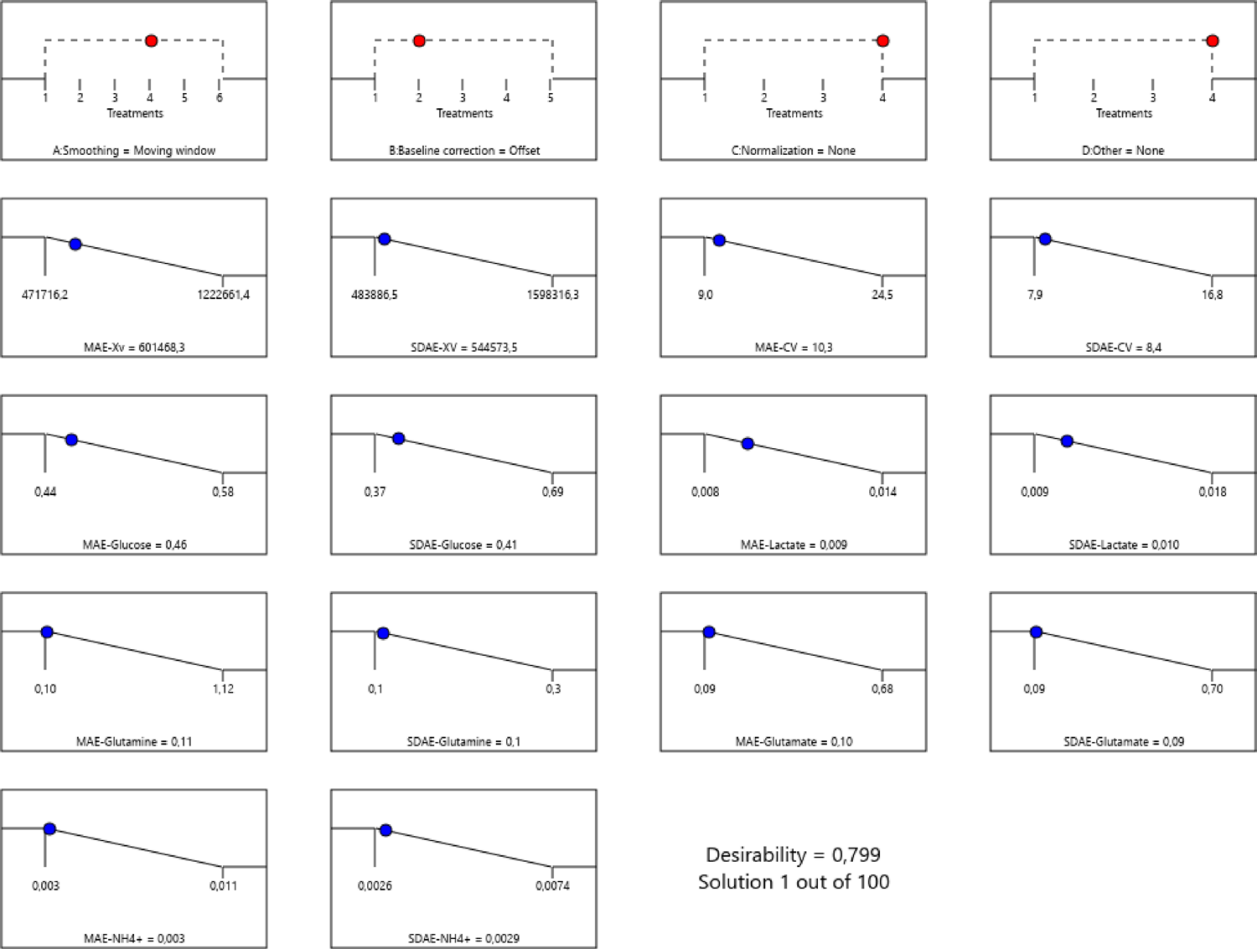
Ramp graph for the optimal combination of spectral filters that simultaneously meets the minimum values of the MAE and SD_AE_ prediction statistical metrics for all combined biochemical parameters under study when PLS modeling was used. Graphs on the first row represent the best result for each pre-treatment from optimization (each number corresponds to the different treatment that could be used). The other graphs bellow represent values for each parameter to achieve the lowest value for MAE and SD_AE_. Dots indicate the best result for each parameter, and desirability corresponds to the analysis of this optimization; better results correspond to values closer to or equal to 1.

When optimizing the combination of filters for all parameters simultaneously, the values of MAE ± SD_AE_ for each biochemical parameter were found to be equal to or higher than those obtained when optimizing the filter combination for each parameter individually (Table 2). Only spectral smoothing filter (Moving window) and baseline correction (Offset) were necessary; normalization and other assessed approaches were not statistically significant after application of numeric optimization using desirability function (Table 2; Fig. 2, Fig. 4A-B).

A-B). This is not a typical preprocessing combination reported for biopharmaceutical applications, where there exists a consensus toward using 1^st^ or 2^nd^ Savitzky-Golay derivative, Savitzky-Golay smoothing (window size varies), SNV to correct for variations in optical scattering [2].

It was also possible to corroborate that for most biochemical parameters, the $\:\mathrm{M}\mathrm{A}\mathrm{E}$ and $\:{\mathrm{S}\mathrm{D}}_{\mathrm{A}\mathrm{E}}$ values approached the minimum values within the ranges for these statistical indicators of prediction quality in all spectral filtering combinations assessed, with Xv and Lactate being the parameters that presented the worst performance (Fig. 2).

The results from this approach to the optimization of $\:\mathrm{M}\mathrm{A}\mathrm{E}$ and $\:{\mathrm{S}\mathrm{D}}_{\mathrm{A}\mathrm{E}}$, including simultaneously all biochemical parameters, even though they are slightly inferior to the alternative of individual parameter optimization in some cases (CV and Glu), are within the error and dispersion ranges for the same host cell, and others frequently used in the pharmaceutical industry [13].

### Models’ fitting for MAE and SDAE using ANN for each biochemical parameter

To perform the optimized choice of the combination of spectral filters that minimizes the $\:\mathrm{M}\mathrm{A}\mathrm{E}$ and $\:{\mathrm{S}\mathrm{D}}_{\mathrm{A}\mathrm{E}}$ for each biochemical parameter individually or considering all simultaneously, statistical modeling was performed using ANN of these two predictive quality indicators of regression models (Supplementary material: Tables 1 and 3). The models presented were referred to the coded independent variables and derived from a complete factorial design as well as executed for PLS.

**Table 3:** Best ANN-generated models for overall and individual optimization. A total of 480 combinations of spectral filters, derived from a database of 65 samples collected across five batch experiments, were used to calibrate the MAE and SDAE models.

Parameter	Optimization analysis	Spectra preprocessing approach					Training correlation coefficient	Test correlation coefficient	Validation correlation coefficient	Hidden activation function	Output activation function	N_HN_	MAE ± SD_AE_
		Smoothing	Baseline correction	Normalization	Other	Number of PC							
Xv (10^5^ cells/mL)	Overall	WDS	AsLS_C	SNV	1^st^ Der. Quad.	17	0.997	0.996	0.995	Exponential	Logistic	15	1.19 ± 1.21
	Individual	WDS	AsLS_C	-	1^st^ Der. Quad.	23	0.997	0.998	0.996	Exponential	Tanh	22	1.11 ± 1.04
CV (%)	Overall	WDS	AsLS_C	SNV	1^st^ Der. Quad.	17	0.989	0.989	0.993	Logistic	Identity	7	3.23 ± 2.81
	Individual	WDS	AsLS_C	-	1^st^ Der. Quad.	23	0.997	0.993	0.993	Tanh	Exponential	21	*1.86 ± 2.05
Gluc (g/L)	Overall	WDS	AsLS_C	SNV	1^st^ Der. Quad.	17	0.907	0.907	0.727	Exponential	Exponential	9	0.30 ± 0.31
	Individual												
Lac (10^− 3^ g/L)	Overall	WDS	AsLS_C	SNV	1^st^Der. Quad.	17	0.987	0.918	0.959	Tanh	Exponential	5	3.50 ± 4.45
	Individual												
Gln (g/L)	Overall	WDS	AsLS_C	SNV	1^st^ Der. Quad.	17	0.968	0.978	0.957	Logistic	Exponential	7	0.06 ± 0.05
	Individual												
Glu (g/L)	Overall	WDS	AsLS_C	SNV	1^st^ Der. Quad.	17	0.930	0.946	0.854	Exponential	Identity	5	0.06 ± 0.06
	Individual												
NH_4_^+^ (10^− 3^ g/L)	Overall	WDS	AsLS_C	SNV	1^st^ Der. Quad.	17	0.982	0.955	0.975	Logistic	Exponential	13	2.05 ± 1.81
	Individual												

ANN: Artificial Neural Network; PC: principal component; N_HN_: number of neurons in the hidden layer; MAE: mean absolute error; SD_AE_: standard deviation of absolute error

^*^MAE from individual optimization analysis is lower than that from overall optimization. Finding defined by t-test

In ANN models for $\:\mathrm{M}\mathrm{A}\mathrm{E}$ and $\:{\mathrm{S}\mathrm{D}}_{\mathrm{A}\mathrm{E}}$, all the statistically significant factors (General spectral filterings: smoothing, baseline correction, normalization, others; Table 1) and interactions significant for the PLS approach were also important, adding the interaction baseline correction and other-spectral-filter category (Supplementary material: Tables 1 and 3). This may cause the predictive capacity of the ANN model to perform better than PLS. In bioprocess using mammalian cell lines, the use of nonlinear-based models, such as ANN, have demonstrated better fitting to the experimental data than the classic PLS approach [14, 15].

### Selection of the best combination of spectral filters for each biochemical parameter in an ensemble mode using ANN

When the filter combination optimization for each parameter was performed, it was possible to define that the most frequent spectral filters for smoothing, baseline correction, normalization, and others were WDS, AsLS, SNV, and 1^st^quadratic derivative, respectively (Table 3). Only the viable cell density (Xv) and cell viability (CV) parameters presented a different optimal combination, which differed in no need for performing spectral normalization. However, a statistically significant difference was confirmed just in the case of CV.

### Selection of the best combination of spectral filters for all biochemical parameters analyzed simultaneously using ANN

**Fig. 3:**
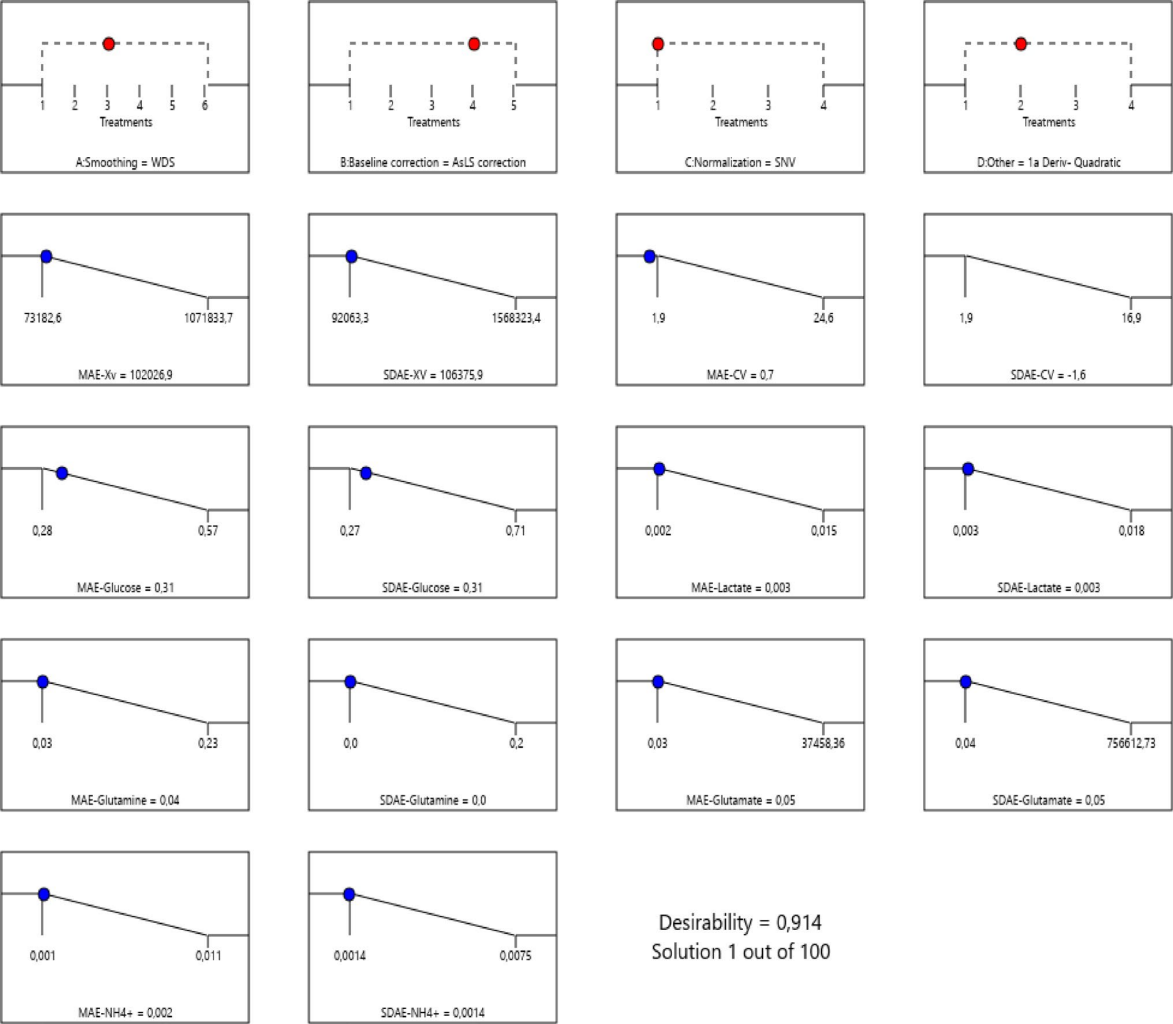
Ramp graph for the optimal combination of spectral filters that simultaneously meets the minimum values of the MAE and SD_AE_ prediction statistical metrics for all combined biochemical parameters under study when ANN modeling was used. Graphs on the first row represent the best result for each pre-treatment from optimization (each number corresponds to the different treatment that could be used). The other graphs bellow represent values for each parameter to achieve the lowest value for MAE and SD_AE_. Dots indicate the best result for each parameter, and desirability corresponds to the analysis of this optimization; better results correspond to values closer to or equal to 1.

**Fig. 4:**
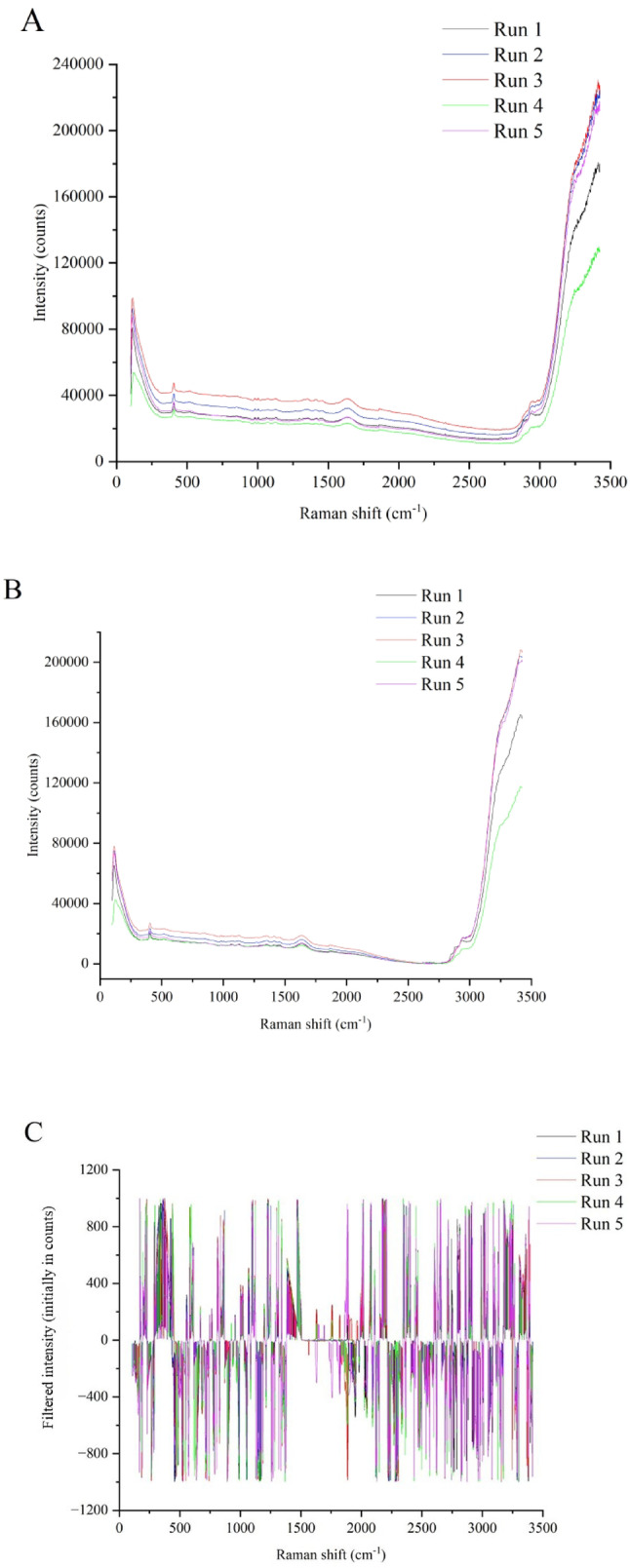
Raw spectrum and corresponding spectral filters with the best prediction performance considering all biochemical parameters simultaneously using PLS and ANN. A: Raw spectra corresponding to samples from the middle of the monitoring time in the five runs. B: Best overall combination of spectral filters in PLS modeling corresponding to samples from the middle of monitoring time in the five runs (Table 2). C: Best overall combination of spectral filters in ANN modeling corresponding to samples from the middle of monitoring time in the five runs (Table 3).

The overall filter combination for the ANN modeling technique, WDS, AsLS, SNV, and 1^st^ quadratic derivative (Table 3; Fig. 3) was similar to individual filter combination optimization for the vast majority of the assessed biochemical parameters. Therefore, this filter combination could be recommended for Raman spectroscopy monitoring of the baculovirus/*Sf9* insect cell line using Sf-900^TM^ III as a culture medium and ANN as a regression tool, because it can reduce the computational and analytical time (Fig. 4).

### Relative error comparison between PLS and ANN best filter combinations

**Fig. 5:**
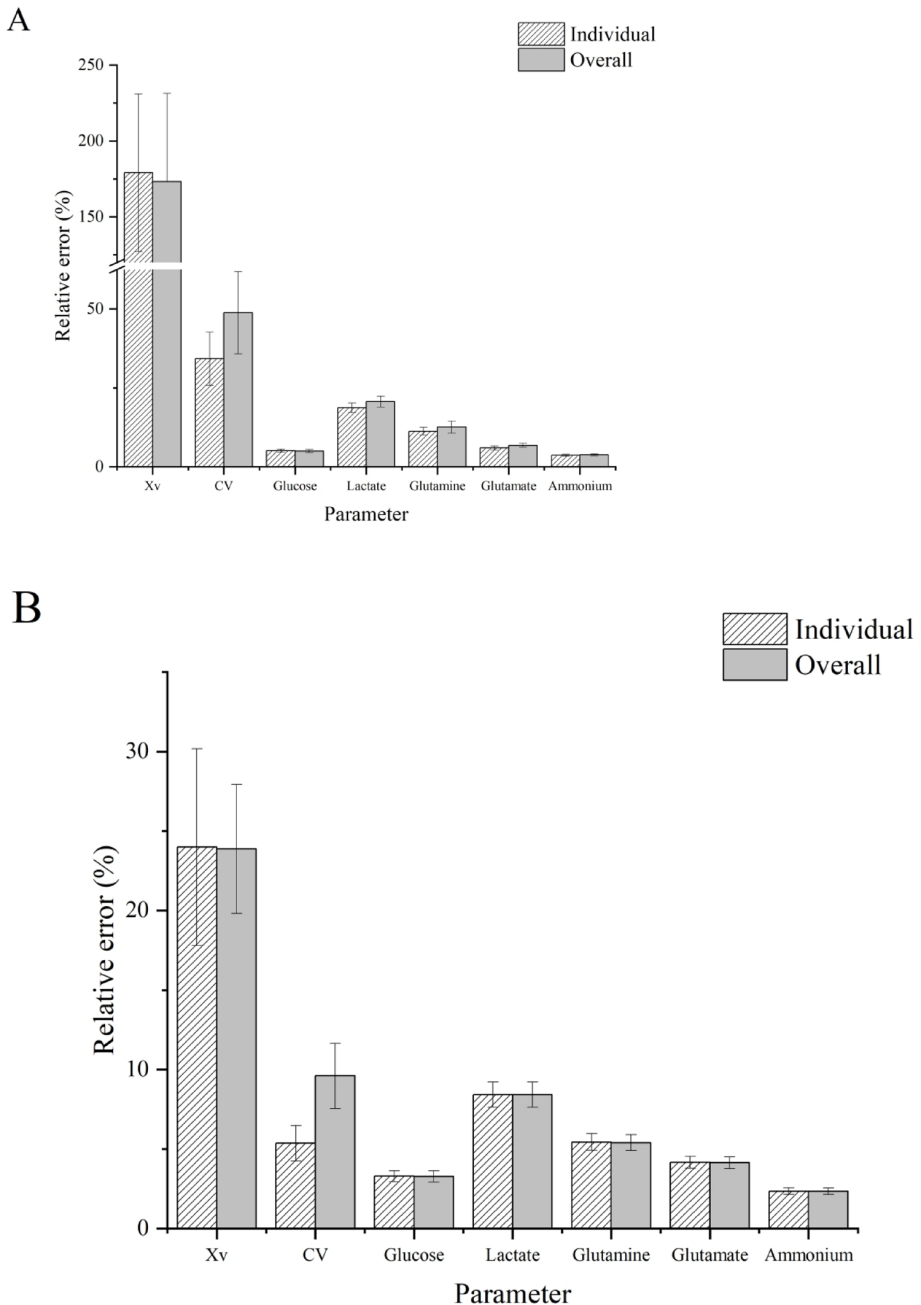
The relative error means in both optimization spectral filters’ combination (individual, overall). The error bars represent the 95% confidence limits for the mean. A: The best PLS models. B: The best ANN models.

The relative error for Xv and CV was higher than that for other biochemical parameters, regardless of the regression technique (Fig. 5).

When t-test comparisons were performed between equivalent optimal filter combinations (individual or overall) using PLS or ANN, no statistical differences were confirmed for relative errors of the biochemical parameters. This could be caused by the wide dispersion of the relative error (95% confidence limits for the mean) among both optimization conditions for all assessed parameters (Fig. 5). However, the average and its 95% confidence interval were lower in ANN than in PLS. Specifically, for ANN, the relative error average was inferior to 10%, except for Xv (24%). Nonlinear methods like ANN offer several benefits, particularly their ability to flexibly model intricate and nonlinear relationships, as a rule, they showed better performance than PLS [16]. Besides, ANN models enhance robustness, and they manage better with inter-batch heterogeneity than PLS models[17].

### Biochemical parameters’ profile simulation

**Fig. 6:**
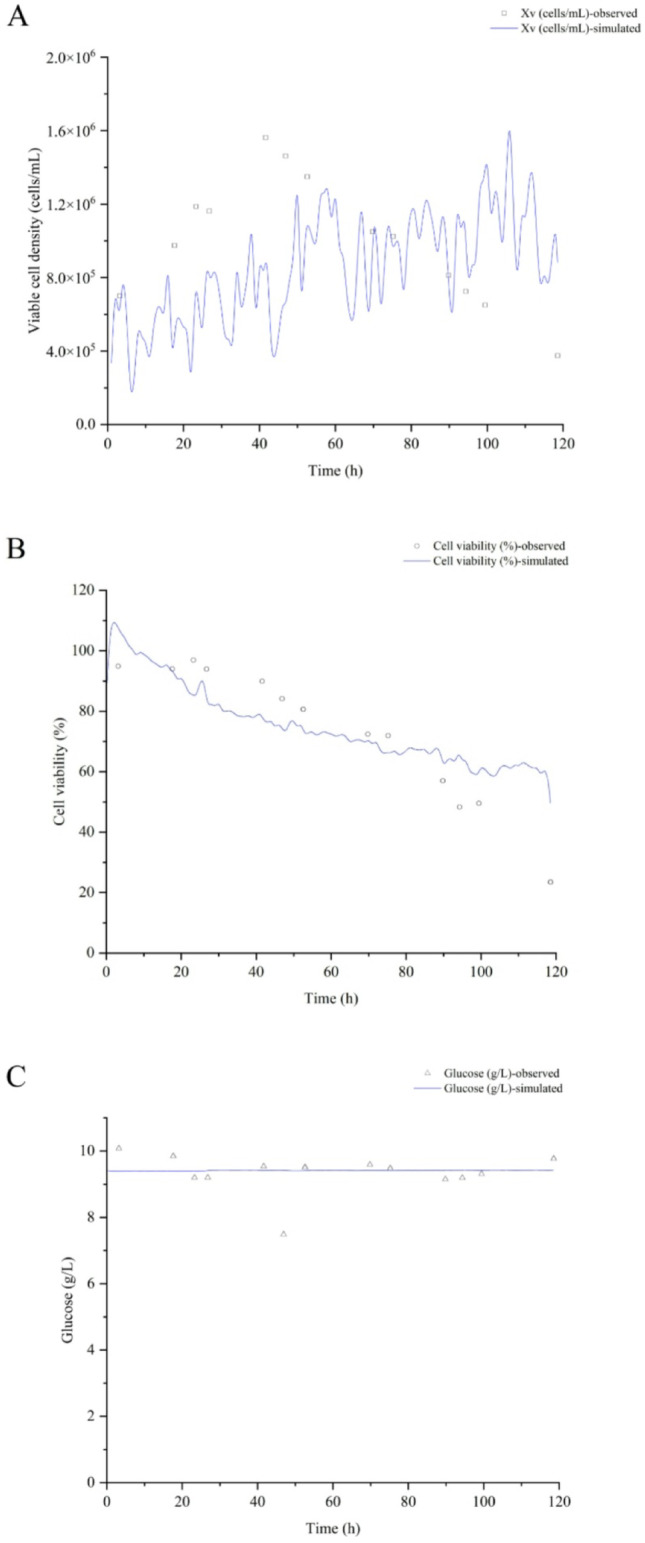

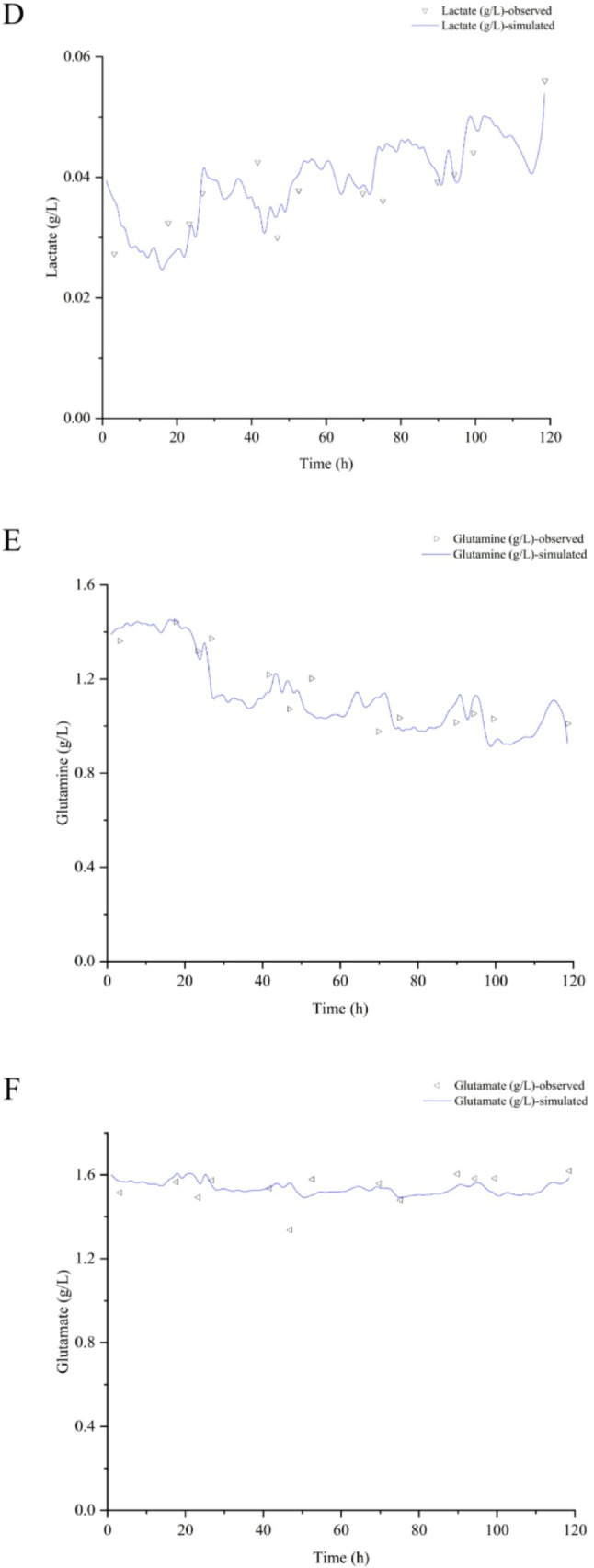

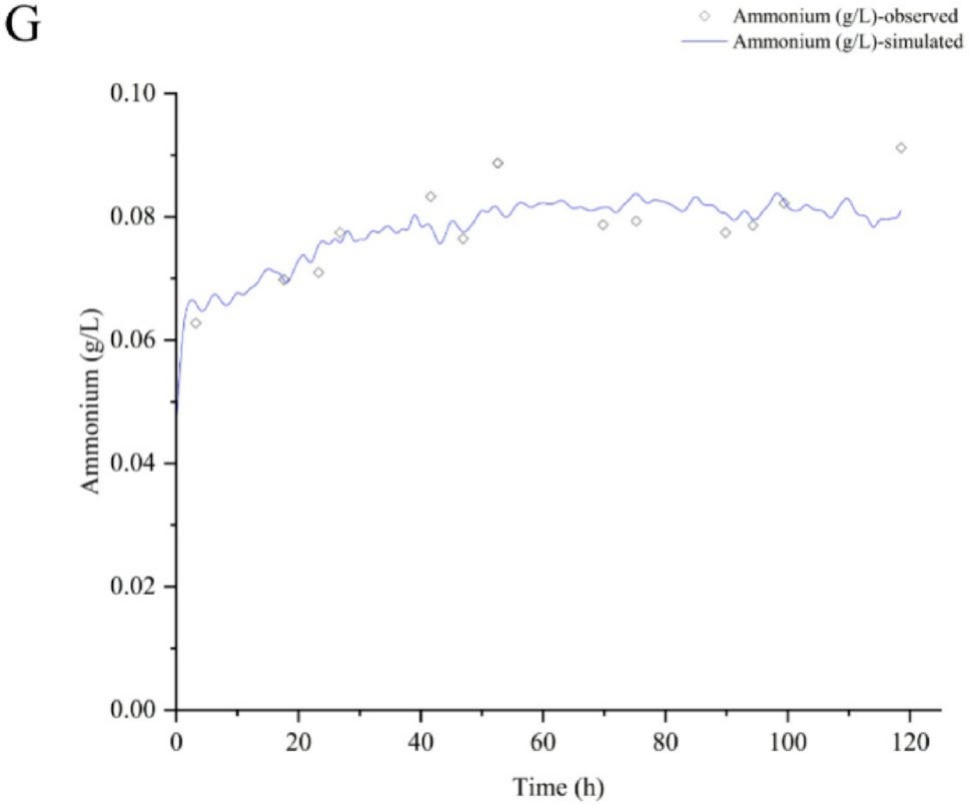
Biochemical parameters’ simulation using the best overall spectral filters combination in PLS modeling for coinfection assay to make rabies VLP (rBV-G: rBV-M, MOI of 3:2). A: Viable cell density (Xv). B: Cell viability (CV). C: Glucose (Gluc). D: Lactate (Lac). E: Glutamine (Gln). F: Glutamate (Glu). G: Ammonium (NH_4_^+^).

The biochemical parameters’ profile simulation was performed using the best overall spectral filters combination in PLS to illustrate the prediction quality of the models in one of the bioreactor assays, by applying the chemometric method that demonstrated relatively reduced predictive performance, and the most established one for this type of application in pharmaceutical bioprocesses. A proper agreement with observed data was found except for Xv (Fig. 6, Supplementary material: Figures 1, 2 and 3), with errors like those reported for biochemical parameters in the Chinese hamster ovary cell line bioprocess [17,18,19 and 20]. The glucose and glutamate drops (close to 20%) around 45 h were not well-predicted by both ANN (data not shown) and PLS. This could be associated with the wave-shaped profiles of nutrients during baculovirus lytic infection mechanism [13].

Errors for viable cell densities higher than 0.5 $\:\times\:$ 10^6^ cell mL^− 1^ have been consistently reported [20]. Therefore, this is an issue for the system focused on in our work, where infection processes occur when a viable cell density of approximately 1 × 10^6^ cell mL^− 1^ is reached.

## Conclusions

To the best of our knowledge, this study is the first to employ statistical methodologies grounded in factorial experimental design to rigorously recommend the optimal combination of spectral filters for Raman-based monitoring in systems utilizing insect cells. The spectral filters’ combination of the moving window smoothing and offset baseline correction showed the best performance in biochemically monitoring the baculovirus/*Sf9* insect cell system based on Raman spectroscopical data and its modeling through PLS. However, another combination of filters turned out to be the most suitable when applying ANN modeling (wavelet denoise spectral smoothing, asymmetric least square baseline correction, standard normal variate normalization, and quadratic first derivate). Thus, these findings could be useful for the community of professionals involved in inline Raman monitoring, carefully guiding which combination of spectral filters can be used depending on the modeling technique used, avoiding the trial-and-error approach.

The simulation results demonstrate that by following these guidelines for spectral preprocessing, cell viability, glucose, lactate, glutamine, glutamate, and ammonium can be satisfactorily quantified in real-time, but not the viable cell density.

## Supplementary Information

Below is the link to the electronic supplementary material.

Supplementary Material 1

## Data Availability

Primary data will be available upon the corresponding author’s request.
