# Supplementary material for: Selecting Raman spectra filtering based on an exhaustive statistical approach for inline bioprocesses monitoring using Sf9 insect cells
Source: Bioprocess Biosyst Eng. 2026 Mar 26;49(4):999–1013. doi: 10.1007/s00449-026-03301-1 (PMC13124868; doi:10.1007/s00449-026-03301-1)

Supplementary material

Manuscript: Selecting Raman spectra filtering based on an exhaustive statistical approach for online bioprocesses monitoring using the baculovirus/insect cell system

Table 1. Relationship between the names of the original variables and the corresponding independent variables used in the polynomial-coded equations for mean absolute error and standard deviation of absolute error to assess the PLS and ANN model predictions for each biochemical parameter using spectral filter combinations in a two-factorial design of experiments.

| Equation variables | Original independent variables |
| --- | --- |
| Intercept |  |
| A[1] | Savitzky-Golay |
| A[2] | EWMA |
| A[3] | WDS |
| A[4] | Moving window |
| A[5] | AsLS Smoothing |
| B[1] | Row-Center |
| B[2] | Offset |
| B[3] | Linear |
| B[4] | AsLS Correction |
| C[1] | SNV |
| C[2] | Peak Height |
| C[3] | Peak Area |
| D[1] | MSC |
| D[2] | 1a Deriv-Quadratic |
| D[3] | 2a Deriv-Cubic |

Table 2. Final equations in terms of coded factors for mean absolute error and standard deviation of absolute error for assessing the PLS model prediction to each biochemical parameter using spectral filters’ combinations according to a two-factorial design of experiments.

| Factors | (MAE-Xv)^-1.38^ | (SDAE-Xv)^-2.69^ | Log₁₀(MAE-CV) | SDAE-CV | MAE-Glucose | SDAE-Glucose | MAE-Lactate | (SDAE- Lactate)^-1.28^ | (MAE-Glutamine)^-2.18^ | (SDAE-Glutamine)^-1.79^ | (MAE-Glutamate)^-2.47^ | (SDAE-Glutamate)^-2.19^ | (MAE-NH_4_^+^)^-1.52^ | (SDAE-NH_4_^+^)^-1.55^ |
| --- | --- | --- | --- | --- | --- | --- | --- | --- | --- | --- | --- | --- | --- | --- |
| **Intercept** | 8.27E-09 | 2.14E-16 | 1.23 | 13.38 | 0.4717 | 0.4272 | 0.011 | 283.24 | 85.33 | 56.76 | 191.26 | 111.03 | 3442.87 | 5487.84 |
| **A[1]** | -4.01E-11 | 6.25E-18 | 0.0435 | 0.6807 | 0.0021 |  | 0.0003 | -15.96 | -5.26 | -3.08 | 6.78 | 6.86 | -526.17 | -747.26 |
| **A[2]** | 4.74E-10 | 3.71E-17 | -0.0061 | 0.1775 | -0.0013 |  | 0.0002 | -0.623 | -5.19 | -2.81 | 4.39 | 5.61 | -181.37 | -184.93 |
| **A[3]** | -3.72E-10 | -6.84E-17 | -0.0082 | -0.7332 | -0.0014 |  | -0.0005 | 11.28 | 12.74 | 5.72 | -41.47 | -30.42 | 984.22 | 1101.26 |
| **A[4]** | 5.01E-10 | 3.90E-17 | -0.0136 | -0.1143 | 0.0023 |  | -0.0003 | 16.1 | 1.82 | 0.5687 | 15.46 | 10.31 | 88 | 272.43 |
| **A[5]** | -1.04E-09 | -5.34E-17 | -0.0109 | -0.2359 | -0.0003 |  | 0.0001 | -10.8 | 2.29 | 2.35 | 9.13 | 0.8919 | -108.43 | -140.08 |
| **B[1]** | -1.77E-10 | 1.50E-18 | -0.0028 | -0.1156 | 0.0024 | 0.009 |  | -0.8259 | 2.45 | 2.36 | -1.37 | 0.8224 | 363.8 | 508.07 |
| **B[2]** | 1.88E-10 | 1.99E-17 | -0.0035 | -0.1914 | -0.0051 | -0.01 | -0.0003 | 14.98 | 4.56 | 3.91 | 3.91 | 4.88 | 465.01 | 703.1 |
| **B[3]** | 3.63E-10 | 2.17E-17 | -0.0047 | -0.2218 | -0.0066 | -0.0028 | 0.0002 | -11.95 | 2.58 | -1.6 | -4.59 | -2.17 | 186.15 | 195.43 |
| **B[4]** | -3.38E-10 | -6.09E-17 | 0.0252 | 0.8918 | 0.0132 | 0.0144 | 0.0005 | -20.21 | -17.31 | -9.63 | -3.9 | -10.79 | -868.06 | -1229.01 |
| **C[1]** |  |  | -0.0105 | -0.2907 | -0.0018 | -0.0074 | -0.0001 | 13.68 |  |  |  | -0.8185 | 31.34 | 100.1 |
| **C[2]** |  |  | 0.0135 | 0.2872 | 0.0034 | 0.01 | 0.0001 | -8.25 |  |  |  | -2.62 | -151.14 | -231.32 |
| **C[3]** |  |  | -0.0024 | -0.0642 | 0.0027 | 0.0053 | -0.0001 | 4.53 |  |  |  | 0.4457 | 118.16 | 205.63 |
| **D[1]** | 1.12E-09 | 6.80E-17 | -0.0389 | -1.15 |  |  | -0.0007 | 36.95 | 24.99 | 12.56 | 0.5468 | 6.95 | 918.4 | 1301.33 |
| **D[2]** | -8.88E-10 | -5.74E-17 | 0.049 | 1.46 |  |  | 0.0005 | -27.56 | -12.8 | -7.17 | -19.26 | -11.46 | -443.11 | -619.01 |
| **D[3]** | -1.33E-09 | -8.08E-17 | 0.057 | 1.51 |  |  | 0.0009 | -41.49 | -33.66 | -18.27 | -25.1 | -21.27 | -1388.69 | -1928.71 |
| **A[1]B[1]** | -6.41E-11 | -5.31E-18 | 0.0006 | 0.0813 | -0.0011 |  | -0.0001 | 7.84 | -6.14 | -0.7662 | 4.46 | 1.64 | 184.88 | 279.93 |
| **A[2]B[1]** | 4.48E-10 | 1.58E-17 | -0.0117 | -0.1992 | 0.0034 |  |  | 1.66 | 3.41 | 2.37 | -3.4 | -3.19 | 69.06 | 83.52 |
| **A[3]B[1]** | -8.52E-10 | -2.16E-17 | 0.053 | 0.9496 | -0.0043 |  | 0.0006 | -31.26 | -11.44 | -9.26 | -14.57 | -6.08 | -299.21 | -420.58 |
| **A[4]B[1]** | 1.79E-10 | 6.80E-18 | -0.0052 | -0.0755 | 0.0025 |  |  | -0.9067 | 3.07 | 0.8625 | 1.15 | -1.48 | -231.69 | -295.62 |
| **A[5]B[1]** | -1.56E-10 | -9.38E-18 | -0.0231 | -0.501 | -0.0039 |  | -0.0004 | 21.59 | 7.04 | 4.54 | 16.87 | 13.26 | 139.06 | 162.18 |
| **A[1]B[2]** | 2.51E-10 | 1.76E-17 | 0.0217 | 0.4959 | -0.0043 |  | -0.0001 | 0.2676 | -12.86 | -1.9 | -7.41 | -4.15 | 44.49 | 24.34 |
| **A[2]B[2]** | 2.06E-10 | 6.27E-18 | -0.0114 | -0.1883 | 0.0013 |  |  | 2.8 | 4.57 | 2.34 | -4.07 | -2.47 | 36.96 | 126.62 |
| **A[3]B[2]** | -6.61E-10 | -2.15E-17 | 0.028 | 0.4042 | 0.0036 |  | 0.0006 | -19.58 | -7.15 | -7.14 | -4.49 | -3.47 | -483.68 | -738.15 |
| **A[4]B[2]** | 2.22E-10 | 1.52E-18 | -0.0203 | -0.4226 | -0.0043 |  | -0.0003 | 11.05 | -1.56 | -1.34 | 5.84 | 1.39 | 321.74 | 489.7 |
| **A[5]B[2]** | -2.22E-10 | -7.77E-18 | -0.005 | -0.0533 | 0.0025 |  | -0.0002 | 3.31 | 11.23 | 5.77 | 15.53 | 12.3 | -31.35 | -145.62 |
| **A[1]B[3]** | 1.60E-10 | -1.18E-18 | -0.0317 | -0.6406 | -0.0035 |  | 0.0002 | -7.67 | -0.373 | -4.82 | 12.23 | -0.188 | 13.53 | 21.21 |
| **A[2]B[3]** | 3.63E-10 | 2.01E-17 | 0.0084 | 0.2359 | 0.0015 |  | -0.0001 | 2.83 | -3.97 | -2.4 | 5.71 | 5.05 | -254.28 | -448.56 |
| **A[3]B[3]** | -1.21E-09 | -3.82E-17 | 0.0184 | 0.603 | 0.006 |  | 0.0002 | -11.57 | -9.54 | -3.12 | -29.81 | -16.22 | -226.63 | -253.76 |
| **A[4]B[3]** | 2.56E-10 | 9.30E-18 | 0.0077 | 0.0213 | -0.0052 |  | 0.0003 | -11.39 | 6.16 | 6.05 | 14 | 17.41 | 424.17 | 643.28 |
| **A[5]B[3]** | 6.72E-11 | -7.65E-18 | -0.0128 | -0.4023 | -0.0005 |  | -0.0004 | 25.6 | 10.5 | 6.74 | -6.51 | -9.96 | 222.61 | 369.9 |
| **A[1]B[4]** | -7.75E-10 | -3.52E-17 | 0.0047 | -0.0805 | 0.0136 |  | 0.0002 | -12.95 | 18.92 | 8.51 | 0.0025 | 10.24 | -679.47 | -920.82 |
| **A[2]B[4]** | -1.22E-09 | -4.87E-17 | 0.0387 | 0.6857 | -0.0069 |  | 0.0003 | -18.25 | -10.98 | -5.83 | -1.02 | -2.26 | -274.48 | -355.02 |
| **A[3]B[4]** | 3.72E-09 | 1.18E-16 | -0.1708 | -3.39 | -0.0103 |  | -0.0022 | 111.06 | 43.18 | 29.25 | 63.1 | 33.64 | 693.29 | 1025.41 |
| **A[4]B[4]** | -8.82E-10 | -2.53E-17 | 0.0337 | 0.841 | 0.0119 |  | 0.0004 | -15.02 | -7.37 | -4.77 | -22.43 | -14.23 | 759.93 | 853.36 |
| **A[5]B[4]** | 3.88E-10 | 4.19E-17 | 0.0568 | 1.32 | -0.0014 |  | 0.0008 | -46 | -33.71 | -21.29 | -37.4 | -24.09 | -293.59 | -354.98 |
| **A[1]C[1]** |  |  | 0.0115 | 0.347 |  |  |  |  |  |  |  |  |  |  |
| **A[2]C[1]** |  |  | -0.0076 | -0.1336 |  |  |  |  |  |  |  |  |  |  |
| **A[3]C[1]** |  |  | -0.0295 | -0.7277 |  |  |  |  |  |  |  |  |  |  |
| **A[4]C[1]** |  |  | 0.0125 | 0.3136 |  |  |  |  |  |  |  |  |  |  |
| **A[5]C[1]** |  |  | 0.0221 | 0.382 |  |  |  |  |  |  |  |  |  |  |
| **A[1]C[2]** |  |  | -0.0078 | -0.1153 |  |  |  |  |  |  |  |  |  |  |
| **A[2]C[2]** |  |  | 0.0239 | 0.5784 |  |  |  |  |  |  |  |  |  |  |
| **A[3]C[2]** |  |  | -0.0177 | -0.3347 |  |  |  |  |  |  |  |  |  |  |
| **A[4]C[2]** |  |  | 0.0134 | 0.2974 |  |  |  |  |  |  |  |  |  |  |
| **A[5]C[2]** |  |  | -0.0343 | -0.9306 |  |  |  |  |  |  |  |  |  |  |
| **A[1]C[3]** |  |  | -0.0018 | -0.0419 |  |  |  |  |  |  |  |  |  |  |
| **A[2]C[3]** |  |  | 0.0026 | 0.1733 |  |  |  |  |  |  |  |  |  |  |
| **A[3]C[3]** |  |  | -0.007 | -0.24 |  |  |  |  |  |  |  |  |  |  |
| **A[4]C[3]** |  |  | -0.0022 | -0.1543 |  |  |  |  |  |  |  |  |  |  |
| **A[5]C[3]** |  |  | 0.0075 | 0.1439 |  |  |  |  |  |  |  |  |  |  |
| **A[1]D[1]** | 1.02E-09 | 7.47E-17 | 0.0815 | 1.57 |  |  | -0.0004 | 18.79 | 12.16 | 9.87 | 34.88 | 26.07 | 356.28 | 400.99 |
| **A[2]D[1]** | 8.90E-10 | 5.49E-17 | -0.0533 | -1.22 |  |  | -0.0006 | 34.22 | 11.81 | 6.42 | 29.13 | 25.92 | 484.27 | 797.92 |
| **A[3]D[1]** | -1.58E-09 | -1.08E-16 | 0.094 | 2.35 |  |  | 0.0018 | -88.8 | -25.91 | -16.54 | -97.24 | -63.87 | -1142.98 | -1583.38 |
| **A[4]D[1]** | 7.26E-10 | 3.08E-17 | -0.0384 | -0.6806 |  |  | -0.0002 | 1.01 | 9.94 | 3.09 | -5.27 | -16.52 | 514.88 | 586.43 |
| **A[5]D[1]** | -1.94E-09 | -1.05E-16 | -0.029 | -0.7483 |  |  |  | 1.22 | -21.07 | -9.19 | 10.78 | 3.69 | -775.56 | -1122.52 |
| **A[1]D[2]** | -9.73E-10 | -6.51E-17 | -0.0354 | -0.5727 |  |  | 0.0004 | -19.62 | -6.48 | -4.55 | -4.92 | -0.6528 | -416.75 | -509.5 |
| **A[2]D[2]** | -7.84E-10 | -5.95E-17 | 0.0237 | 0.7028 |  |  | 0.0005 | -25.17 | -13.25 | -6.49 | 1.64 | -3.89 | -335.93 | -555.42 |
| **A[3]D[2]** | 1.10E-09 | 8.07E-17 | -0.0605 | -1.66 |  |  | -0.0014 | 73.33 | 15.15 | 9.86 | -21.57 | -5.56 | 586.42 | 774.7 |
| **A[4]D[2]** | -5.55E-10 | -3.68E-17 | 0.0059 | 0.1874 |  |  | 0.0003 | -7.41 | -13.95 | -6.42 | 4.26 | 2.33 | -354.91 | -289.2 |
| **A[5]D[2]** | 2.00E-09 | 1.43E-16 | 0.0445 | 0.7129 |  |  | -0.0003 | 4.79 | 30.47 | 14.15 | 20.51 | 12.98 | 769.71 | 998.9 |
| **A[1]D[3]** | -2.58E-10 | -3.09E-17 | 0.0032 | -0.2042 |  |  | 0.0003 | -12.35 | -10.07 | -7.4 | -9.65 | -5.28 | -393.27 | -534.3 |
| **A[2]D[3]** | -6.58E-10 | -4.49E-17 | 0.0092 | 0.5924 |  |  | 0.0002 | -11.38 | -11.04 | -5.24 | -12.87 | -12.03 | -716.38 | -1050.03 |
| **A[3]D[3]** | 1.33E-09 | 1.12E-16 | -0.047 | -1.37 |  |  | -0.0011 | 51.44 | 33.15 | 18.54 | 46.42 | 33.47 | 1427.93 | 2009.53 |
| **A[4]D[3]** | -5.76E-10 | -2.28E-17 | 0.0231 | 0.2785 |  |  | 0.0002 | -2.77 | -11.77 | -3.31 | -4.74 | 3.74 | -485.76 | -759.21 |
| **A[5]D[3]** | 8.24E-10 | 3.43E-17 | 0.0018 | 0.1678 |  |  | 0.0004 | -12.82 | 9.46 | 2.71 | -4.83 | -6.6 | 796.46 | 1248.1 |
| **B[1]D[1]** |  | 1.41E-17 | 0.0119 | 0.1568 |  |  | -0.0006 | 31.77 | 3.72 | 3.26 |  |  | 128.08 | 299.59 |
| **B[2]D[1]** |  | -4.33E-18 | 0.0126 | 0.2327 |  |  | -0.0003 | 15.97 | -2.8 | 0.6101 |  |  | 118.44 | 229.4 |
| **B[3]D[1]** |  | 1.72E-17 | -0.0527 | -1.28 |  |  | 0.0008 | -46.83 | 1.27 | -4.69 |  |  | 216.63 | 105.78 |
| **B[4]D[1]** |  | -2.46E-17 | 0.0051 | 0.4832 |  |  | 0.0003 | -13.84 | -3.46 | -0.4633 |  |  | -70.97 | -186.44 |
| **B[1]D[2]** |  | -2.08E-18 | -0.0006 | 0.0991 |  |  | 0.0001 | -4.29 | 0.7193 | 1.05 |  |  | 3.08 | -39.83 |
| **B[2]D[2]** |  | 6.96E-18 | 0.011 | 0.3785 |  |  | 0 | -0.1179 | 1.09 | 0.9149 |  |  | -91.95 | -203.11 |
| **B[3]D[2]** |  | -2.15E-17 | 0.0069 | 0.0902 |  |  | 0 | 3.74 | -5.22 | -1.66 |  |  | -168.15 | -116.58 |
| **B[4]D[2]** |  | 2.07E-17 | -0.03 | -0.9371 |  |  | -0.0004 | 19.7 | 4.68 | 2.12 |  |  | -43.53 | 57.38 |
| **B[1]D[3]** |  | -1.57E-17 | 0.0055 | 0.1547 |  |  | 0.0002 | -9.75 | -4.69 | -4.04 |  |  | -64.21 | -87.5 |
| **B[2]D[3]** |  | -6.39E-18 | -0.0018 | 0.0542 |  |  | 0.0003 | -13.46 | -6.18 | -4.46 |  |  | -380.25 | -621.53 |
| **B[3]D[3]** |  | -1.75E-17 | -0.0035 | 0.0672 |  |  | -0.0004 | 25.32 | -0.1158 | 3.89 |  |  | -340.58 | -351.8 |
| **B[4]D[3]** |  | 4.98E-17 | -0.0166 | -0.7119 |  |  | -0.0006 | 24.05 | 18.12 | 9.68 |  |  | 618.46 | 880.4 |
| **C[1]D[1]** |  |  | 0.0096 | 0.2676 |  |  | 0.0001 | -12.74 |  |  |  | 2.34 | -38.28 | -122.82 |
| **C[2]D[1]** |  |  | -0.0109 | -0.2189 |  |  | -0.0002 | 8.55 |  |  |  | -1.9 | 239.23 | 398.12 |
| **C[3]D[1]** |  |  | 0.0015 | 0.0417 |  |  | 0.0001 | -6.91 |  |  |  | 1.38 | -120.3 | -228 |
| **C[1]D[2]** |  |  | 0.0064 | 0.0991 |  |  | 9.49E-06 | -2.13 |  |  |  | 2.78 | -188.08 | -430.71 |
| **C[2]D[2]** |  |  | -0.0391 | -0.9487 |  |  | -0.0003 | 11.24 |  |  |  | 6.42 | -431.65 | -588.99 |
| **C[3]D[2]** |  |  | 0.0002 | 0.2725 |  |  |  | -1.3 |  |  |  | -0.6343 | 344.85 | 487.17 |
| **C[1]D[3]** |  |  | -0.0159 | -0.3358 |  |  | 0.0002 | -14.46 |  |  |  | 0.8653 | -38.98 | -91.17 |
| **C[2]D[3]** |  |  | -0.0297 | -0.5371 |  |  | -0.0003 | 21.96 |  |  |  | 5.83 | -45.5 | -23.72 |
| **C[3]D[3]** |  |  | -0.0015 | -0.1983 |  |  | 0.0001 | -2.18 |  |  |  | -2.51 | 148.14 | 205.1 |

Table 3. Final equations in terms of coded factors for mean absolute error and standard deviation of absolute error for assessing the ANN model prediction to each biochemical parameter using spectral filters’ combinations according to a two-factorial design of experiments.

| Factors | Log₁₀(MAE-Xv) | (SDAE-XV)^-0,5^ | (MAE-CV)^0,5^ | SDAE-CV | MAE-Glucose | (SDAE-Glucose)^-2,21^ | Log₁₀(MAE-Lactate) | Log₁₀(SDAE-Lactate) | Log₁₀(MAE-Glutamine) | (SDAE-Glutamine)^-0,5^ | (MAE-Glutamate)^-1^ | (SDAE-Glutamate)^-1^ | (MAE-NH_4_^+^)^-0,5^ | (SDAE-NH_4_^+^)^-0,5^ |
| --- | --- | --- | --- | --- | --- | --- | --- | --- | --- | --- | --- | --- | --- | --- |
| **Intercept** | 5.58 | 0.0016 | 3.28 | 10.26 | 0.4306 | 8.18 | -2.13 | -2.07 | -0.9862 | 3.41 | 11.3 | 11.57 | 16.41 | 17.91 |
| **A[1]** | 0.034 | -0.0001 | 0.1539 | 0.4058 | 0.0259 | -0.8943 | 0.0049 | 0.0364 | 0.0323 | -0.1297 | -0.7779 | -0.3705 | -0.7943 | -0.6891 |
| **A[2]** | 0.059 | -0.0001 | 0.268 | 1.16 | 0.0005 | -0.0174 | 0.0367 | 0.043 | 0.0567 | -0.1888 | -0.6621 | -0.6658 | -1.12 | -1.09 |
| **A[3]** | -0.1534 | 0.0004 | -0.7783 | -3.7 | -0.0249 | 0.4921 | -0.1045 | -0.1216 | -0.1505 | 0.5526 | 2.35 | 2.34 | 2.96 | 2.88 |
| **A[4]** | 0.0069 | -0.0001 | 0.0902 | 0.7684 | -0.0064 | 0.239 | 0.0261 | 0.0147 | 0.0335 | -0.1213 | -0.1887 | -0.5571 | -0.565 | -0.6264 |
| **A[5]** | -0.0115 |  | -0.0533 | 0.1034 | 0.0062 | -0.0061 | -0.0039 | -0.0034 | -0.0227 | 0.0512 | -0.1519 | 0.0633 | 0.6655 | 0.6714 |
| **B[1]** | 0.0182 |  | -0.0303 | -0.1834 | 0.0001 | -0.1362 | 0.0015 | 0.0038 | -0.0009 | -0.006 | -0.0236 | 0.0941 | 0.0401 | 0.1312 |
| **B[2]** | -0.0384 | 0.0001 | -0.204 | -1.11 | -0.0093 | 0.3561 | -0.0441 | -0.0407 | -0.0541 | 0.1828 | 0.6771 | 0.787 | 0.8853 | 0.8867 |
| **B[3]** | 0.0194 | -0.0001 | -0.0148 | -0.1367 | 0.0063 | -0.5535 | 0.0249 | 0.0168 | 0.0071 | -0.0482 | -0.438 | -0.6302 | 0.2205 | 0.3425 |
| **B[4]** | 0.0257 |  | 0.4301 | 2.39 | 0.0131 | -0.2829 | 0.0253 | 0.039 | 0.0808 | -0.243 | -0.5931 | -0.6866 | -1.87 | -2.13 |
| **C[1]** | -0.019 |  | -0.1042 | -0.4729 | -0.0132 | 0.5297 | -0.024 | -0.0171 | -0.0257 | 0.0792 | 0.397 | 0.4846 | 0.6612 | 0.5324 |
| **C[2]** | 0.0231 | -0.0001 | 0.0056 | -0.1139 | 0.0118 | -0.4046 | 0.0143 | 0.0185 | 0.0061 | -0.0357 | -0.3635 | -0.5463 | -0.3646 | -0.1305 |
| **C[3]** | 0.0224 | -0.0001 | 0.1253 | 0.5687 | 0.0056 | -0.2502 | 0.0318 | 0.0248 | 0.0258 | -0.0939 | -0.2632 | -0.3892 | -0.3399 | -0.2713 |
| **D[1]** | -0.0755 | 0.0002 | -0.5482 | -2.94 | -0.0189 | 0.4312 | -0.0601 | -0.0627 | -0.0908 | 0.3038 | 1.02 | 0.9101 | 2.18 | 2.12 |
| **D[2]** | 0.0258 | -0.0001 | 0.3425 | 1.83 | 0.0134 | -0.0149 | 0.0319 | 0.0381 | 0.0367 | -0.1532 | -0.4958 | -0.3076 | -0.775 | -0.68 |
| **D[3]** | 0.1259 | -0.0003 | 0.6654 | 3.49 | 0.0251 | -0.7908 | 0.0858 | 0.102 | 0.1311 | -0.4188 | -1.63 | -1.61 | -3.22 | -3.18 |
| **A[1]B[1]** | 0.0535 | -8.85E-06 | 0.0743 | 0.1291 | -0.0036 | 0.272 | -0.0057 | 0.0062 | -0.0025 | -0.0053 | -0.1248 | -0.3788 | -0.3623 | -0.1667 |
| **A[2]B[1]** | -0.079 | 0.0001 | -0.1314 | -0.4942 | -0.0043 | -0.0948 | -0.0054 | -0.003 | -0.0135 | 0.0524 | 0.2321 | 0.2804 | 0.7663 | 0.5982 |
| **A[3]B[1]** | 0.0826 | -0.0002 | 0.402 | 1.77 | 0.0318 | -1.06 | 0.0606 | 0.052 | 0.0799 | -0.3205 | -1.47 | -1.18 | -1.38 | -1.28 |
| **A[4]B[1]** | 0.0105 |  | -0.1009 | -0.1373 | -0.0102 | 0.5148 | -0.0025 | -0.0083 | -0.0161 | 0.083 | 0.2321 | 0.2071 | 0.1396 | 0.1885 |
| **A[5]B[1]** | -0.0348 |  | -0.0695 | -0.4998 | -0.0038 | -0.0349 | -0.0209 | -0.0263 | -0.0192 | 0.0481 | 0.5581 | 0.4721 | 0.0631 | 0.0803 |
| **A[1]B[2]** | -0.0089 | -4.64E-06 | 0.0851 | 0.0769 | 0.0019 | -0.1935 | -0.0286 | -0.0081 | 0.0049 | -0.0304 | -0.161 | -0.2584 | -0.239 | -0.0206 |
| **A[2]B[2]** | 0.0012 | -7.33E-06 | 0.0132 | 0.18 | -0.0068 | 0.5776 | -0.0039 | 0.0052 | -0.0085 | 0.0343 | 0.56 | 0.5509 | 0.2652 | 0.133 |
| **A[3]B[2]** | 0.0732 | -0.0001 | 0.1795 | 0.9268 | 0.0201 | -0.8097 | 0.0598 | 0.0465 | 0.0524 | -0.1769 | -0.9612 | -0.9125 | -1.06 | -1.05 |
| **A[4]B[2]** | -0.0145 | 0.0001 | -0.0312 | -0.1084 | -0.0077 | 0.0477 | 0.005 | -0.0065 | -0.0114 | 0.0646 | 0.0447 | -0.0151 | 0.3127 | 0.2934 |
| **A[5]B[2]** | -0.0382 | 0.0001 | -0.1646 | -0.7559 | 0.0019 | 0.0401 | -0.0082 | -0.0038 | -0.0251 | 0.0849 | 0.2353 | 0.0866 | 0.1435 | 0.2316 |
| **A[1]B[3]** | -0.0144 | 0.0001 | -0.1043 | -0.2116 | -0.0052 | 0.3653 | 0.0066 | -0.01 | 0.0094 | -0.0031 | 0.294 | 0.3734 | -0.0498 | -0.051 |
| **A[2]B[3]** | 0.007 |  | -0.1019 | -0.7165 | -0.0003 | 0.0715 | -0.0026 | 0.0045 | -0.0118 | 0.0406 | 0.3423 | 0.1771 | 0.1588 | 0.305 |
| **A[3]B[3]** | 0.0404 | -0.0001 | 0.245 | 1.31 | 0.0039 | -0.0014 | 0.0417 | 0.0289 | 0.0363 | -0.1189 | -0.6939 | -0.7698 | -0.6444 | -0.8101 |
| **A[4]B[3]** | -0.0754 | 0.0001 | -0.1916 | -0.9175 | -0.0007 | -0.0502 | -0.0575 | -0.0563 | -0.0243 | 0.0281 | 0.317 | 0.6323 | 0.5938 | 0.6476 |
| **A[5]B[3]** | 0.0469 | -0.0001 | 0.273 | 1.48 | 0.0056 | -0.5698 | 0.01 | 0.0116 | 0.0073 | 0.0006 | -0.4608 | -0.538 | -0.1908 | -0.1591 |
| **A[1]B[4]** | -0.0303 | -0.0001 | 0.0052 | 0.3238 | 0.0047 | -0.3013 | 0.0307 | 0.0191 | 0.0065 | -0.0085 | 0.006 | 0.435 | 0.4973 | 0.2031 |
| **A[2]B[4]** | 0.094 | -0.0001 | 0.3324 | 1.52 | 0.0285 | -1.25 | 0.034 | 0.0181 | 0.0488 | -0.1978 | -1.75 | -1.88 | -1.48 | -1.45 |
| **A[3]B[4]** | -0.2988 | 0.0006 | -1.29 | -6.38 | -0.0771 | 3.19 | -0.2407 | -0.214 | -0.2601 | 0.9702 | 4.74 | 4.44 | 5.31 | 5.39 |
| **A[4]B[4]** | 0.1086 | -0.0003 | 0.3789 | 1.62 | 0.0278 | -0.95 | 0.0664 | 0.0804 | 0.072 | -0.2697 | -0.9622 | -0.7292 | -1.64 | -1.67 |
| **A[5]B[4]** | 0.061 | -0.0001 | 0.2661 | 1.29 | -0.0053 | 0.4232 | 0.0604 | 0.0582 | 0.073 | -0.272 | -0.9106 | -0.8565 | -0.8484 | -0.9762 |
| **A[1]C[1]** |  |  |  |  |  |  |  |  |  |  |  |  |  |  |
| **A[2]C[1]** |  |  |  |  |  |  |  |  |  |  |  |  |  |  |
| **A[3]C[1]** |  |  |  |  |  |  |  |  |  |  |  |  |  |  |
| **A[4]C[1]** |  |  |  |  |  |  |  |  |  |  |  |  |  |  |
| **A[5]C[1]** |  |  |  |  |  |  |  |  |  |  |  |  |  |  |
| **A[1]C[2]** |  |  |  |  |  |  |  |  |  |  |  |  |  |  |
| **A[2]C[2]** |  |  |  |  |  |  |  |  |  |  |  |  |  |  |
| **A[3]C[2]** |  |  |  |  |  |  |  |  |  |  |  |  |  |  |
| **A[4]C[2]** |  |  |  |  |  |  |  |  |  |  |  |  |  |  |
| **A[5]C[2]** |  |  |  |  |  |  |  |  |  |  |  |  |  |  |
| **A[1]C[3]** |  |  |  |  |  |  |  |  |  |  |  |  |  |  |
| **A[2]C[3]** |  |  |  |  |  |  |  |  |  |  |  |  |  |  |
| **A[3]C[3]** |  |  |  |  |  |  |  |  |  |  |  |  |  |  |
| **A[4]C[3]** |  |  |  |  |  |  |  |  |  |  |  |  |  |  |
| **A[5]C[3]** |  |  |  |  |  |  |  |  |  |  |  |  |  |  |
| **A[1]D[1]** | 0.0195 |  | -0.0887 | -0.2713 | 0.0135 | -0.0706 | -0.009 | -0.0024 | -0.0267 | 0.1034 | -0.531 | -0.5442 | 0.318 | 0.2287 |
| **A[2]D[1]** | -0.0533 | 0.0001 | -0.191 | -0.7767 | -0.0276 | 0.8061 | -0.0097 | -0.0121 | -0.0277 | 0.0938 | 0.4076 | 0.4866 | 0.9988 | 0.963 |
| **A[3]D[1]** | 0.0666 | -0.0001 | 0.3349 | 1.47 | 0.02 | -0.6209 | 0.0651 | 0.0601 | 0.0871 | -0.2969 | -1.48 | -1.54 | -1.8 | -1.65 |
| **A[4]D[1]** | 0.0132 | 0.0001 | 0.0854 | 0.1084 | 0.0088 | -0.7384 | -0.0004 | 0.0067 | -0.0349 | 0.139 | 0.3826 | -0.0571 | 0.544 | 0.2427 |
| **A[5]D[1]** | 0.0328 | -0.0002 | 0.1728 | 0.7323 | 0.0073 | 0.008 | 0.0167 | 0.0052 | 0.0635 | -0.2674 | 0.1195 | 0.4475 | -1.13 | -0.6727 |
| **A[1]D[2]** | -0.0091 | -0.0001 | 0.1629 | 0.8113 | 0.0137 | -0.9981 | 0.0119 | 0.0148 | 0.0013 | -0.0355 | -0.0102 | 0.1523 | 0.0288 | 0.1143 |
| **A[2]D[2]** | 0.0667 | -0.0002 | 0.2219 | 1.2 | 0.0189 | -0.4221 | 0.0267 | 0.0547 | 0.0586 | -0.2406 | -0.8341 | -0.7537 | -0.9764 | -0.9479 |
| **A[3]D[2]** | -0.1398 | 0.0004 | -0.695 | -3.36 | -0.0185 | 0.6415 | -0.1036 | -0.1064 | -0.1071 | 0.4526 | 2.24 | 2.35 | 1.79 | 1.92 |
| **A[4]D[2]** | 0.0374 | -0.0001 | 0.0811 | 0.4573 | -0.004 | 0.6084 | 0.0145 | 0.0112 | 0.0428 | -0.1778 | -0.5035 | -0.5902 | -0.3576 | -0.2981 |
| **A[5]D[2]** | -0.0429 | 0.0002 | 0.0122 | -0.0369 | -0.0111 | -0.2213 | -0.0143 | -0.0292 | -0.0569 | 0.2576 | 0.2581 | -0.0079 | 0.6472 | 0.2431 |
| **A[1]D[3]** | -0.0076 | 9.21E-08 | 0.1419 | 0.1787 | -0.0107 | 1.01 | -0.013 | -0.0144 | 0.0308 | -0.1149 | 0.6451 | 0.8835 | -1.16 | -0.9686 |
| **A[2]D[3]** | 0.0661 | -0.0001 | 0.2754 | 0.9396 | 0.021 | -0.9105 | 0.0548 | 0.0404 | 0.04 | -0.1064 | -0.5554 | -0.9499 | -1.06 | -0.9479 |
| **A[3]D[3]** | -0.0412 | 0.0001 | -0.1978 | -0.6425 | -0.0201 | 0.457 | -0.0401 | -0.0524 | -0.0773 | 0.2158 | 0.3378 | 0.2089 | 2.17 | 1.92 |
| **A[4]D[3]** | -0.0414 |  | -0.0153 | 0.5396 | -0.0033 | 0.5401 | -0.0031 | -0.0103 | 0.0298 | -0.0972 | -0.229 | 0.1435 | -0.7359 | -0.5086 |
| **A[5]D[3]** | -0.0345 | 0.0001 | -0.4372 | -1.95 | -0.0082 | -0.0504 | -0.0376 | -0.0097 | -0.066 | 0.2171 | 0.4267 | 0.4452 | 1.76 | 1.41 |
| **B[1]D[1]** | -0.042 | 0.0001 | -0.3324 | -1.81 | -0.0087 | 0.5046 | -0.0522 | -0.0519 | -0.0575 | 0.1786 | 0.7345 | 0.6451 | 1.13 | 1.07 |
| **B[2]D[1]** | -0.0113 | 4.62E-06 | -0.1461 | -0.9281 | 0.0029 | 0.0493 | 0.0008 | 0.0085 | -0.008 | 0.031 | 0.1345 | 0.129 | 0.1416 | 0.2286 |
| **B[3]D[1]** | 0.0299 |  | 0.0614 | 0.5718 | -0.0014 | -0.4931 | 0.0287 | 0.0219 | 0.0408 | -0.1284 | -0.0177 | -0.121 | -0.0509 | 0.1886 |
| **B[4]D[1]** | 0.0486 | -0.0001 | 0.5454 | 3.05 | 0.0088 | -0.007 | 0.0393 | 0.0173 | 0.066 | -0.2132 | -1.29 | -1.12 | -1.81 | -1.93 |
| **B[1]D[2]** | 0.0161 |  | 0.0904 | 0.7239 | 0.007 | -0.4705 | 0.0128 | 0.0276 | 0.0162 | -0.0645 | -0.6757 | -0.7356 | -0.9656 | -0.9812 |
| **B[2]D[2]** | 0.0124 |  | 0.1264 | 0.6557 | 0.0076 | -0.2536 | 0.0104 | 0.0185 | 0.0141 | -0.0643 | -0.5743 | -0.4059 | -0.3274 | -0.1527 |
| **B[3]D[2]** | -0.0085 | -9.13E-06 | 0.1293 | 0.2848 | -0.0075 | 0.6644 | 0.001 | -0.0111 | 0.0011 | 0.0061 | -0.5177 | -0.2955 | -0.367 | -0.4107 |
| **B[4]D[2]** | -0.0307 | 0.0001 | -0.4331 | -2.14 | -0.0141 | 0.4687 | -0.0194 | -0.0254 | -0.0422 | 0.149 | 1.75 | 1.34 | 1.49 | 1.35 |
| **B[1]D[3]** | 0.0445 | -0.0001 | 0.2117 | 1 | 0.009 | -0.1942 | 0.0361 | 0.0311 | 0.0436 | -0.1554 | -0.6285 | -0.748 | -0.5703 | -0.5742 |
| **B[2]D[3]** | 0.0348 | -0.0001 | 0.1824 | 1.23 | 0.0042 | 0.142 | 0.0154 | 0.0117 | 0.0425 | -0.146 | -0.7009 | -0.8139 | -0.604 | -0.7585 |
| **B[3]D[3]** | -0.0338 | 0.0001 | -0.0916 | -0.3112 | -0.0055 | 0.1292 | -0.0161 | -0.0092 | -0.0371 | 0.1472 | 0.7116 | 0.8124 | -0.072 | -0.0814 |
| **B[4]D[3]** | -0.0952 | 0.0002 | -0.5327 | -3.29 | -0.0201 | 0.4829 | -0.0685 | -0.0718 | -0.0928 | 0.3055 | 1.31 | 1.43 | 2.15 | 2.38 |
| **C[1]D[1]** |  |  |  |  | 0.0143 |  | 0.0181 |  |  |  |  |  |  |  |
| **C[2]D[1]** |  | 0.0001 |  |  | -0.0152 |  | -0.0065 |  |  |  |  |  |  |  |
| **C[3]D[1]** |  |  |  |  | -0.0053 |  | -0.0421 |  |  |  |  |  |  |  |
| **C[1]D[2]** |  |  |  |  | 0.0048 |  | -0.0244 |  |  |  |  |  |  |  |
| **C[2]D[2]** |  |  |  |  | 0.0056 |  | 0.0002 |  |  |  |  |  |  |  |
| **C[3]D[2]** |  |  |  |  | -0.0051 |  | 0.0279 |  |  |  |  |  |  |  |
| **C[1]D[3]** |  |  |  |  | -0.0057 |  | 0.012 |  |  |  |  |  |  |  |
| **C[2]D[3]** |  |  |  |  | -0.0084 |  | -0.0067 |  |  |  |  |  |  |  |
| **C[3]D[3]** |  |  |  |  | 0.012 |  | -0.0083 |  |  |  |  |  |  |  |

Figure 1. Biochemical parameters’ simulation using the best overall spectral filters combination in PLS modeling for *Sf9* cell line growth assay without infection. A: Viable cell density (Xv). B: Cell viability (CV). C: Glucose (Gluc). D: Lactate (Lac). E: Glutamine (Gln). F: Glutamate (Glu). G: Ammonium (NH_4_^+^).





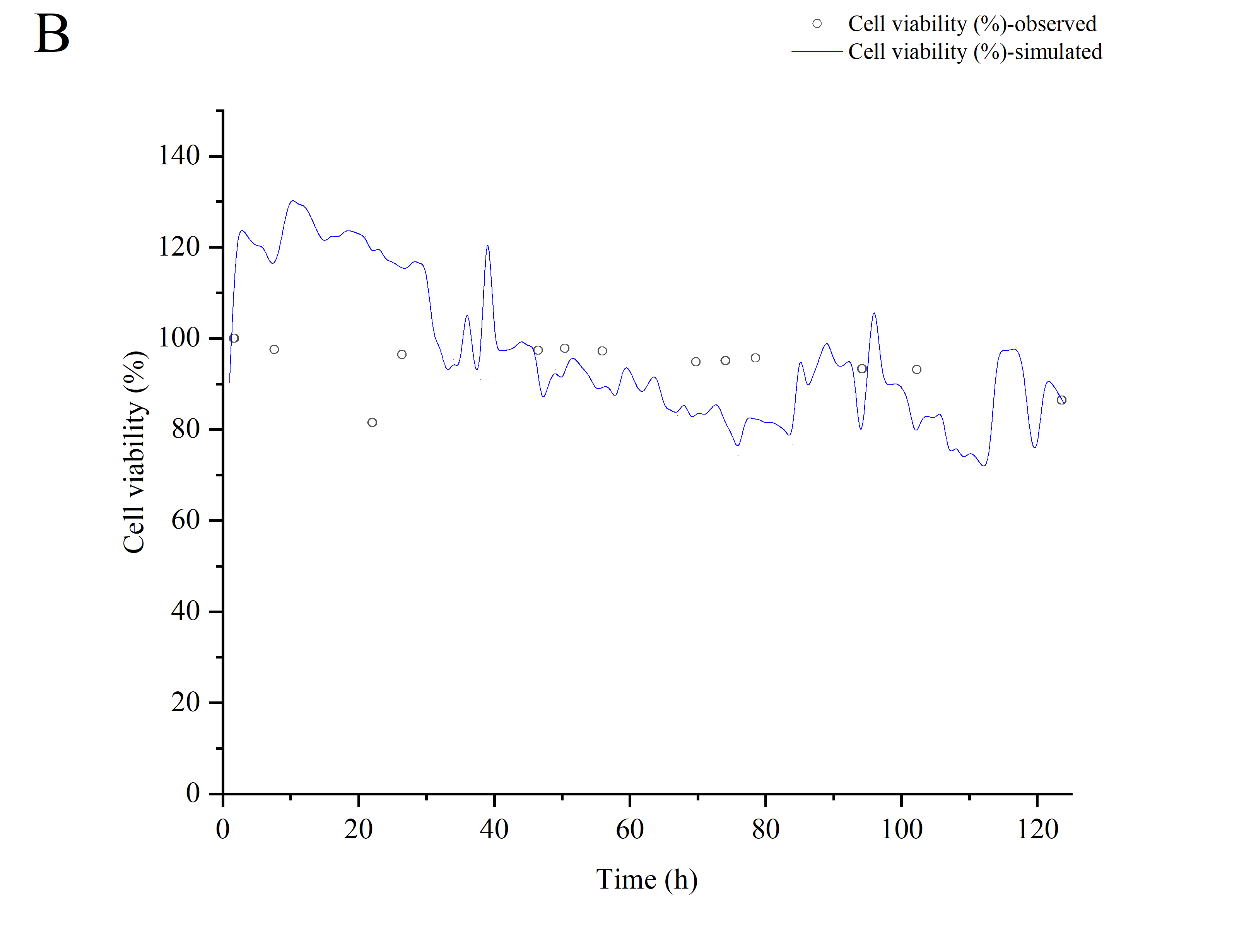

















Figure 2. Biochemical parameters’ simulation using the best overall spectral filters combination in PLS modeling for infection assay to propagate rBV-G, MOI=0.1. A: Viable cell density (Xv). B: Cell viability (CV). C: Glucose (Gluc). D: Lactate (Lac). E: Glutamine (Gln). F: Glutamate (Glu). G: Ammonium (NH_4_^+^).























Figure 3. Biochemical parameters’ simulation using the best overall spectral filters combination in PLS modeling for infection assay to propagate rBV-M, MOI=0.1. A: Viable cell density (Xv). B: Cell viability (CV). C: Glucose (Gluc). D: Lactate (Lac). E: Glutamine (Gln). F: Glutamate (Glu). G: Ammonium (NH_4_^+^).





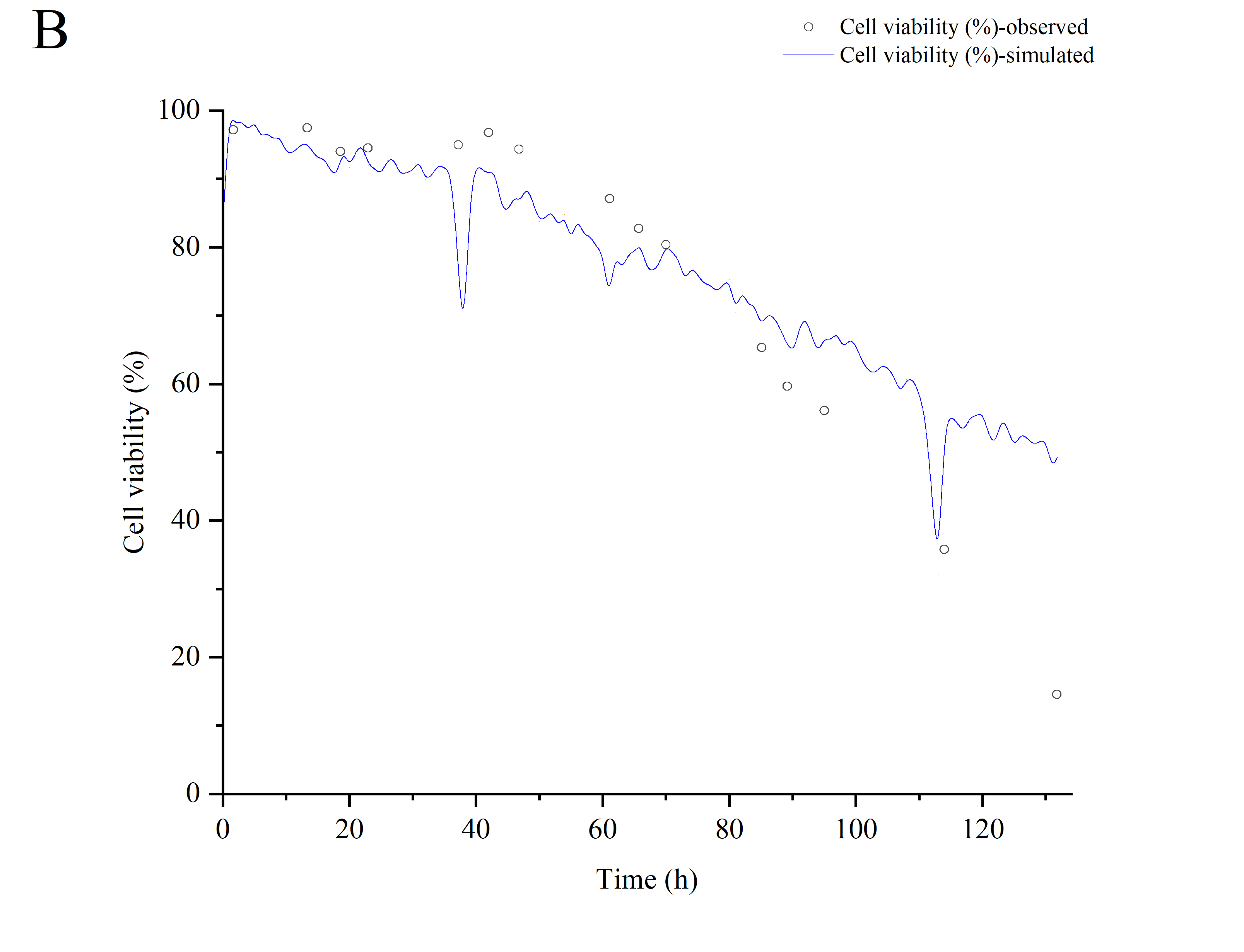

Supplement: Supplementary file 1 — Supplementary Material 1 [file 449_2026_3301_MOESM1_ESM.docx]
